# De novo prediction of *cis*-regulatory elements and modules through integrative analysis of a large number of ChIP datasets

**DOI:** 10.1186/1471-2164-15-1047

**Published:** 2014-12-02

**Authors:** Meng Niu, Ehsan S Tabari, Zhengchang Su

**Affiliations:** Department of Bioinformatics and Genomics, College of Computing and Informatics, The University of North Carolina at Charlotte, 9201 University City Blvd, Charlotte, NC 28223 USA

**Keywords:** *cis*-regulatory elements, *cis*-regulatory modules, ChIP-chip, ChIP-seq, *Drosophila melanogaster*

## Abstract

**Background:**

In eukaryotes, transcriptional regulation is usually mediated by interactions of multiple transcription factors (TFs) with their respective specific *cis*-regulatory elements (CREs) in the so-called *cis*-regulatory modules (CRMs) in DNA. Although the knowledge of CREs and CRMs in a genome is crucial to elucidate gene regulatory networks and understand many important biological phenomena, little is known about the CREs and CRMs in most eukaryotic genomes due to the difficulty to characterize them by either computational or traditional experimental methods. However, the exponentially increasing number of TF binding location data produced by the recent wide adaptation of chromatin immunoprecipitation coupled with microarray hybridization (ChIP-chip) or high-throughput sequencing (ChIP-seq) technologies has provided an unprecedented opportunity to identify CRMs and CREs in genomes. Nonetheless, how to effectively mine these large volumes of ChIP data to identify CREs and CRMs at nucleotide resolution is a highly challenging task.

**Results:**

We have developed a novel graph-theoretic based algorithm DePCRM for genome-wide *de novo* predictions of CREs and CRMs using a large number of ChIP datasets. DePCRM predicts CREs and CRMs by identifying overrepresented combinatorial CRE motif patterns in multiple ChIP datasets in an effective way. When applied to 168 ChIP datasets of 56 TFs from *D. melanogaster*, DePCRM identified 184 and 746 overrepresented CRE motifs and their combinatorial patterns, respectively, and predicted a total of 115,932 CRMs in the genome. The predictions recover 77.9% of known CRMs in the datasets and 89.3% of known CRMs containing at least one predicted CRE. We found that the putative CRMs as well as CREs as a whole in a CRM are more conserved than randomly selected sequences.

**Conclusion:**

Our results suggest that the CRMs predicted by DePCRM are highly likely to be functional. Our algorithm is the first of its kind for *de novo* genome-wide prediction of CREs and CRMs using larger number of transcription factor ChIP datasets. The algorithm and predictions will hopefully facilitate the elucidation of gene regulatory networks in eukaryotes. All the predicted CREs, CRMs, and their target genes are available at http://bioinfo.uncc.edu/mniu/pcrms/www/.

**Electronic supplementary material:**

The online version of this article (doi:10.1186/1471-2164-15-1047) contains supplementary material, which is available to authorized users.

## Background

Since the completion of sequencing the first metazoan genomes in 1998 [1], more than 311 important metazoan and plant genomes have been sequenced thus far [2], and enormous efforts have been made to understand how biological functions and diseases of these organisms including the humans can be explained by the genetic information stored in the genome sequences. Although significant progress has been made in the past 16 years, we are still far from the goal of understanding the biology of metazoans and plants solely from their genome sequences [3]. In fact, it turns out that interpreting a genome is more difficult and challenging than originally thought when a few eukaryotic genomes including the human genome were first released [3, 4]. With this recognition, the community has taken a more realistic approach by first identifying all the functional sequence elements in the genomes [5–7]. These functional elements include transcribed sequences as well as transcriptional control elements, epigenetic features, and regulatory elements acting at the RNA level post-transcriptionally. In principle, while the transcribed sequences specify the potential part list in the cells in an organism, including proteins, various types of RNAs and metabolites, the transcriptional control elements including promoters, enhancers, silencers and insulators together with epigenetic remodeling machineries, determine which protein- or RNA-specifying sequences should be transcribed in each cell during development and under various physiological conditions, thereby specifying the cell’s type during development and specific physiological functions, as it is the dynamic interactions of these components in a cell that determine the cell’s type and specific physiological functions [8]. Once these functional elements are at least partially known, then we can move toward to the next step to identify dynamic interactions among the functional sequence elements and their products of proteins, RNAs and metabolites in different cell types in the entire life of the organism.

In the past we have gained a good understanding of transcribed sequences, particularly protein-coding sequences in numerous sequenced eukaryotic genomes thanks to the development of powerful computational and experimental methods for their characterization [9]. However, we have had only very limited understanding of transcriptional control elements, particularly promoters, enhancers and silencers in virtually all sequenced large eukaryotic genomes, even though these elements are as important as the transcribed sequences for the functions of an organism [10–12]. More specifically, promoters, enhancers and silencers are clusters of closely located *cis*-regulatory elements (CREs) that are recognized by specific transcription factors (TFs) [13]. Thus, a CRE is also called a TF binding site (In this paper, we will refer to a set of similar CREs recognized by the same TF as a *motif*). These clusters of CREs are also called *cis*-regulatory modules (CRMs) [13]. The difficulty to identify CREs and CRMs either computationally or experimentally is due mainly to their short and degenerate nature while they mainly reside in very long intergenic or intronic background sequences [14]. To further confound the problem, they can be very far away from the target genes or even can be located on a different chromosome [15], making their characterization extremely difficult by computational methods such as comparative genomics approaches, although there are successful examples, in particular for developmental enhancers that tend to be more conserved [16, 17].

However, in the past a few years, the development of a plethora of next-generation sequencing (NGS)-based high throughput techniques has largely changed the way to characterize CREs or even CRMs genome-wide in large eukaryotic genomes. These techniques include ChIP-chip and ChIP-seq for locating CREs of a TF [18–20] and various chromatin modification marks [21], DNase-seq [22–24] and FAIRE-seq [23] for locating free nucleosome regions which tend to coincide with active CRMs, and Hi-C for measuring the physical proximity of linearly distal DNA segments [25, 26]. While a single epigenetic dataset derived from DNase-seq [22–24], FAIRE-seq [23] or enhancer mark ChIP-seq potentially contains location information of all CRMs active in a cell or tissue type, CREs and CRMs for specific TFs cannot be easily identified in such a dataset, as it lumps all CREs and CRMs active in the cell or tissue type. In contrast, a TF ChIP-seq dataset is highly enriched for the CREs of the TF, thus they can be potentially identified at single nucleotide resolution in a cell or tissue or type. However, the sequenced potential binding regions in a TF ChIP-seq dataset can be still much longer than the CREs of the ChIP-ed TF, thus peak-calling algorithms and tools have been developed to identify the binding peaks in the potential binding regions. Even though the existing peak-calling algorithms can narrow down CREs of a ChIP-ed TF to a certain regions, typically from a few hundred to a few thousand base pairs (bp) [27], they are still much longer than the typical lengths of CREs, which are typically 6 ~ 16 bp long. Hence, the actual locations of CREs need to be identified by a motif-finding tool [28, 29]. Although a few new motif-finders have been developed to analyze large sequence sets from ChIP-seq experiments, such as seeder [30], Trawler [30, 31], ChIPMunk [32], HMS [33], CMF [34], STEME [35], DREME [36], DECOD [37], RSAT [38], and POSMO [39], they are typically used to find the CREs of a ChIP-ed TF in a short region of sequences (~200 bp) around the binding peak summits in order to reduce the searching space and increase prediction specificity in trading of sensitivity. Some of these tools [33, 39] use the locations of binding peaks to help find the CREs of a ChIP-ed TF. Thus only CREs of the ChIP-ed TF are returned by these tools. However, CREs in higher eukaryotes rarely work alone, instead, they cooperate with one another by forming CRMs for combinatorial regulations [13]. It has been shown that CREs of cooperative TFs of a ChIP-ed TF can be found in the neighborhoods of the binding peaks of the ChIP-ed TF [40–44]. In this sense, the information of CREs in a ChIP dataset is not fully explored by the majority of current studies that were mainly targeted to identify the CREs of a ChIP-ed TF.

With the continuous drop in costs of NGS technologies, TF ChIP-seq is becoming routine in numerous individual labs worldwide, and enormous ChIP-seq datasets are being produced in many important metazoans and plants, in addition to the large amount of ChIP data churned out by large consortiums such as the ENCODE [5, 45] and modENCODE [6] projects aimed at identifying all the functional sequence elements in the genomes of humans and the model organisms *C. elegans* [43] and *D. melanogaster* [42, 46]. It is highly expected that very soon, at least one ChIP-seq dataset will be available in a certain cell type, tissue or developmental stage for the majority of TFs encoded in the genomes through these efforts. Since certain combinations of TFs are often repeatedly used for regulating one or more groups (regulons) of genes in some cell types, tissues and developmental stages [10], the increasing number of ChIP-seq datasets contains a wealth of information about the combinatorial patterns of different TFs for transcriptional regulation [42, 43]. Thus, it is now possible to predict the CREs and CRMs genome-wide through integrating the information about co-occurrence of motifs in a large number of ChIP-seq datasets for different TFs from different cell types, tissues, developmental stages and physiological conditions. Although a few methods such as SpaMo [40], CPModule [41] and [47], have been made to identify CREs of cooperator TFs in a ChIP-seq dataset, they do not integrate multiple ChIP-seq datasets, and cannot predict novel motifs in CRMs, as they all depend on a library of known CREs such as TRANSFAC [48] or JASPAR [49] to scan for possible cooperative CREs in binding peaks. Consequently, simple and approximate methods were often used to find motifs in big ChIP datasets. For instance, in recent studies using the modENCODE [42] and ENCODE [50, 51] datasets, only the top 250 and 500 binding peaks with a length of 100 bp and 200 bp, respectively, in each dataset were used to find motifs. Hence, the wealthy information in the valuable ChIP datasets was not fully explored.

In this paper, we have developed a new algorithm DePCRM for genome-wide [*de*] *novo* [p]rediction of [CRMs] and CREs by identifying overrepresented patterns of motif combinations in a large number of ChIP datasets in a sequenced eukaryotic organism. When applied to the *D. melanogaster* genome using a total of 168 ChIP-chip and ChIP-seq datasets for 56 TFs, DePCRM identified 184 CRE motifs and 115,932 CRMs, recovering 77.9% of known CRMs located in the datasets and 89.3% of known CRMs containing at least one predicted CRE. Thus the algorithm has achieved rather high prediction accuracy even using this limited number of datasets.

## Results

### Basic idea of the algorithm

**Figure 1:**
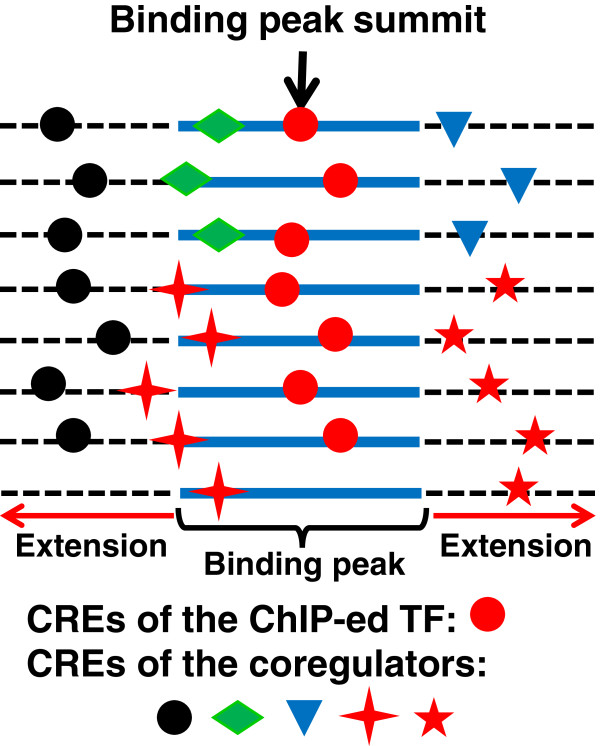
**A schematic view of our hypothesis.** If the binding peak is shorter than 3,000 bp, we equally extended from the two ends to have a length up to 3,000 bp. We assume that in addition to the CREs of the ChIP-ed TF (red circle), CREs of different cooperative TFs (the other shapes) are also enriched in the neighborhoods of at least some subsets of the binding peak dataset. Each line represents an extended binding peak sequence.

As TFs in eukaryotes tend to work together by binding to their CREs in CRMs with a typical size of 500 ~ 3,000 bp [52], we assume that although a ChIP experiment is mainly aimed to identify the binding locations of the ChIP-ed TF, if we extend shorter binding peaks toward the two ends to reach the typical size of CRMs (e.g., 3,000 bp), then extended binding peaks are more likely to contain the CREs of different cooperative TFs (TFs that co-act in a CRM) in addition to the CREs of the ChIP-ed TF as illustrated in Figure 1. In other words, if two different TFs (e.g. the red circle and black circle TFs in Figure 1) cooperatively regulate the same regulons in certain cell types by binding to their respective CREs in CRMs, then their extended ChIP binding peaks from these cell types should overlap with one another to some extent. Hence, if we have enough number of ChIP datasets for different TFs from the same and/or different cell types, then the datasets are likely to include overlapping binding peaks for cooperative TFs. Accordingly, our algorithm predicts CRMs through identifying overrepresented co-occurring putative motif patterns in a large number of ChIP datasets, ideally for different TFs in different cell types and developmental stages.

**Figure 2:**
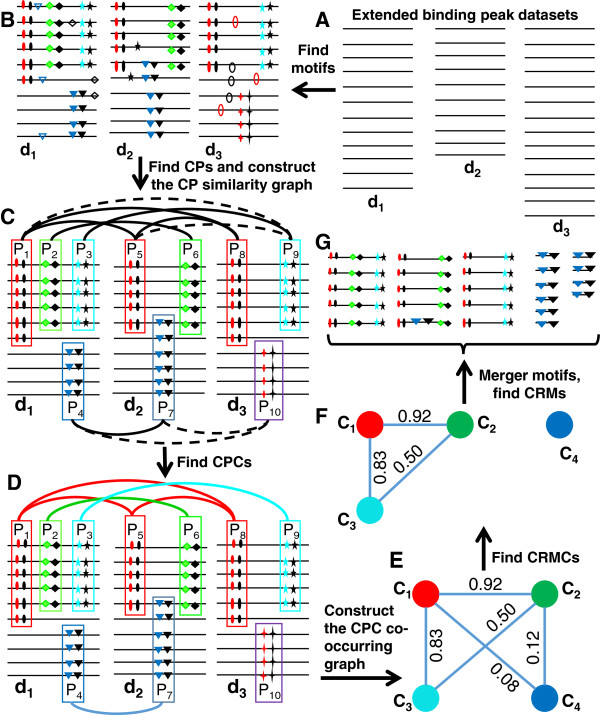
**A schematic of the major steps of the DePCRM algorithm. A**. Illustration of extended binding peaks from dataset d_1_, d_2_ and d_3_ respectively. **B**. Illustration of CREs found within each dataset, CREs of the same motif are shown in the same shape and color. **C**. Construction of CP similarity graph. {P_1_, P_2_, P_3_, P_4_}, {P_5_, P_6_, P_7_} and {P_8_, P_9_, P_10_} are sets of CPs found in datasets d_1_, d_2_ and d_3_ respectively. For clarity, the CPs formed between motifs in P_1_ and motifs in P_2_ and so on in the datasets are not shown. Each CP (represented as a rectangle) is a node of the multi-partied similarity graph, and two nodes are linked by an edge if and only if their *S*_*s*_ ≥ *β*, with *S*_*s*_ being the weight, which is not shown for clarity. **D**. By removing the dotted edges in panel C, MCL cuts the graph into five CP clusters (CPCs): C_1_ = {P_1_, P_5_, P_8_}; C_2_ = {P_2_, P_6_}, C_3_ = {P_3_, P_9_} , C_4_ = {P_4_, P_7_)} and C_5_ = {P_10_}. CPs in a cluster are connected by edges in the same color. The singleton cluster C_5_ = {P_10_} is discarded for its low density. **E**. For each pair C_i_ and C_j_ from the four CPCs, we find sets of CPs from the same dataset *d*_*k*_, and compute a co-occurring scores *S*_*CPC*_ (C_i_, C_j_) for the two CPCs. **F**. Construction of the CPC co-occurring graph using the four CPCs. Cutting the graph using MCL results in two CRMCs, {C1,C2 ,C3} and {C4}. **G**. After merging motifs into Unique motifs (Umotifs), we project the CREs of CRMCs to the genome and predict the CRMs.

More specifically, first, we identify all possible motifs in each of extended binding peak datasets (Figure 2A and B) using a fast motif finder. Second, we find overrepresented co-occurring motif pairs regardless of their distance in each of the datasets, and call them *co-occurring pairs* (CPs) (Figure 2B and C). Third, we reason that if some highly similar CPs appear in multiple datasets, then all these similar CPs are likely to be subsets of the motifs of two certain TFs that cooperatively regulate regulons in different cell types or developmental stages, and therefore are likely to form CRMs by themselves or to be a part of larger CRMs. We identify such repeatedly occurring similar CPs in multiple datasets, and call them *CP clusters* (CPCs) (Figure 2D). Presumably, each of the CPCs contains highly similar CPs for two certain TFs. Fourth, to predict CRMs containing more than two CREs, we cluster CPCs if they tend to co-occur in the same binding peaks (Figure 2E). Each CPC cluster corresponds to a possible combination of their motifs to form a part of or an entire CRM dependent on the sufficiency of the datasets, and thus we refer to them as *CRM components* (CRMCs) (Figure 2F). Finally, we predict individual CRMs across the genome based on the motif pattern of the CRMCs and their close adjacency (Figure 2G). Obviously, in order to accurately predict CRMs genome-wide, we need to have a sufficiently large number of diverse TF ChIP datasets, so that they likely include datasets for cooperative TFs in different cell types and developmental stages. We expect that the more complete the datasets, the more accurate the predictions will be. The details of the algorithm are described in Methods.

### Overlap of the extended binding peaks of cooperative TFs in the datasets

**Figure 3:**
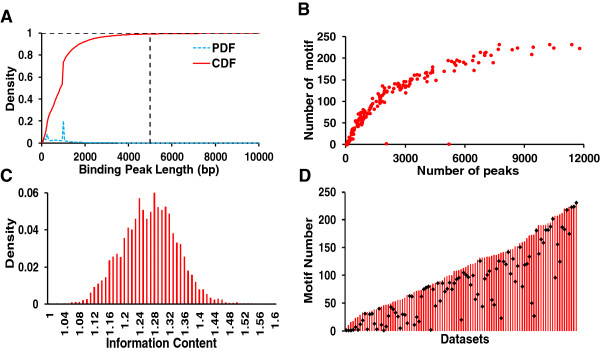
**Analysis of the original datasets and motif finding results. A**. Distribution of the binding peak lengths in the 168 original datasets. Vast majority (99.38%) of the binding peaks are shorter than 5,000 bp. **B**. Number of motifs found in each of the 168 datasets as a function of the number of binding peaks the datasets. **C**. Distribution of the information content of the predicted motifs in the datasets. **D**. The rank of the ChIP-ed TF’s motif among the predicted motifs in the 99 datasets in which the motifs of the ChIP-ed TFs can be identified. The diamond on the bar indicates the rank of the ChIP-ed TF’s motif among the predicted motifs in the dataset. The higher the position of the diamond, the higher the rank of the target TF’s motif.

**Figure 4:**
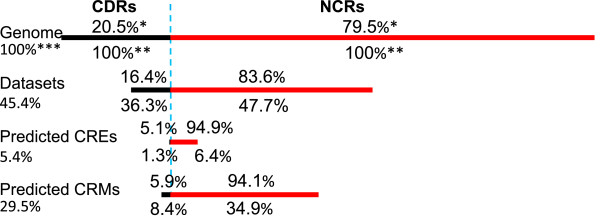
**Coverage of the datasets, predicted CREs and CRMs on the genome and its CDRs and NCRs.** *, the numbers above a line (sequence category) are the percentages of the CDRs and NCRs in the category. **, the numbers below a line are the percentages of CDRs and NCRs of the category with respect to the entire CDRs and NCRs in the genome. ***, the number on the right of a line is the percentage of the category with respect to the genome.

Since *D. melanogaster* has been long used to study gene transcriptional regulation in metazoans, a relatively large number of its CREs and CRMs have been experimentally characterized, and since a large number of ChIP-chip and ChIP-seq have been generated in the organism in the last few years, we evaluated our algorithm in this organism. To this end, we compiled a total of 168 ChIP-seq and ChIP-chip datasets for 56 distinct TFs, collected at different developmental stages (embryo, larva stage 1–3, pupa and adult female and male) and under different experimental conditions (heat shock and etc.). More specifically, 42 ChIP-chip and 42 ChIP-seq datasets were from the modENCODE project [42, 46], 38 Chip-chip datasets were from the Berkeley Drosophila Transcription Network Project (BDTNP) [53], and 46 ChIP-chip datasets were from literature. Additional file 1: Table S1 summarizes the major features of the 168 datasets. As shown in Figure 3A, the majority of the binding peaks have a length around 1,000 bp, and only 0.62% of them have a length longer than 5,000 bp, which were not used in our study due to their low quality. Furthermore, if a binding peak is shorter than 3,000pb, we extended it up to 3,000pb (Methods) in order to include CREs of possible cooperative TFs (Figure 1). The datasets contain a total of 445,252 sequences, each individual dataset containing 26 to 11,772 sequences (Additional file 2: Figure S8). These 445,252 sequences contain a total of 1,183,049,646 bp, which are 7.0 times of the genome (168,736,537 bp), but only cover 45.4% (76,555,033 bp) of the genome (Additional file 3: Table S2), indicating that some of these sequences highly overlap with one another, thus confirming our aforementioned assumption. Of the 76,555,033 bp genome sequence covered by the datasets, 64,033,300 bp (86.3%) are in non-coding regions (NCRs, including introns and intergenic sequences), consisting of 47.7% of NCRs (134,207,178 bp) in the genome (Figure 4 and Additional file 3: Table S2). The remaining 12,521,733 (16.4%) sequences are in coding regions (CDRs), consisting of 36.3% of CDRs (34,529,359 bp) in the genome (Additional file 3: Table S2 and Figure 4). Thus we have included a considerable portion of CDRs in the datasets, because some binding peaks are located in CDRs. Currently, there are 1,830 known CRMs in *D. melanogaster* in the REDfly database [54], and 1,330 (72.7%) of which are located in the extended binding peaks. We will evaluate our algorithm for its ability to recover these 1,330 known CRMs in the extended binding peaks.

**Figure 5:**
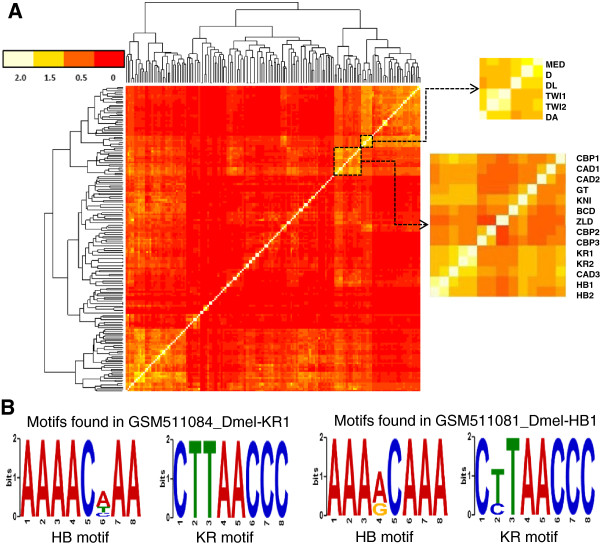
**Overlapping analysis of the datasets. A**. Hierarchical clustering of the 168 datasets for 56 TFs based on their pair-wise binding peak overlapping scores *S*_*o*_. The blow-ups show two clusters for cooperative TFs (see Results). **B**. The motifs of TFs KR and HB are both found in the overlapping datasets GSM511084_Dmel-KR1 ChIP-ed by KR and GSM511081_Dmel-HB1 ChIP-ed by HB.

To see the overlapping patterns of binding peaks upon which our algorithm is based, we computed pair-wise overlapping scores (formula (1) in Methods) of the extended binding peaks among the 168 datasets for the 56 TFs (Additional file 1: Table S1), and clustered the datasets using the overlapping scores. As shown in Figure 5A, consistent with the above analysis, there are significant overlaps among the binding peaks in even these limited 168 datasets for only 5.3% (56/1,052) of the 1,052 annotated TFs encoded in the genome (flytf.org). As expected there are overlaps among datasets of the same TFs collected at differently developmental stages and/or under different experimental conditions, indicating that these TFs might function similarly under these circumstances. For example, the datasets 2625 and 2626 from the modENCODE project were collected using the same TF Caudal (CAD) at the embryonic stages 0–4 hours and adult female, respectively, and they have an overlapping score of 0.5. On the other hand, there are also numerous overlaps among datasets of different TFs. Interestingly, the datasets of TFs that are known to work cooperatively form clusters. The two highlighted boxes in Figure 5A show two examples of such clusters. The upper cluster is formed by the binding peaks for TFs Medea (MED), Dichaete (D), Dorsal (DL), Twist (TWI) and Daughterless (DA). It has been reported that DL and TWI cooperatively regulate the expression of Snail (SNA) in the mesoderm of the embryo [55]. The lower cluster is formed by the binding peaks of the global regulator CREB-binding protein (CBP), gap regulators Kruppel (KR), Giant (GT), CAD and Hunch back (HB). It has been well documented that these TFs bind to CRMs (enhancers/silencers) of genes involved in the segmentation process of early embryogenesis of *D. melanogaster* [54]. An example of such CRMs is shown in Additional file 4: Figure S1. To further evaluate the overlaps of the binding peaks of distinct TFs, we analyzed the 56 out of the 168 datasets, each being for a different TF (if there are multiple datasets of a TF, we selected the one with the largest size), and the same conclusion can be drawn about the overlaps of the binding peaks of different TFs (Additional file 5: Figure S2). The similar results also were reported in *D. melanogaster* [42] and human [56] datasets. Thus these results validate our assumption of the overlaps of binding peaks, and indicate that the datasets might contain sufficient information to predict at least portion of CRMs in the genome.

### Identification of motifs in the extended binding peaks

Our goal now is to identify in each of the extended binding peak datasets all possible TF binding motifs of the ChIP-ed TFs as well as of its cooperative TFs (Figures 1, 2A and B). Because accurate motif-finding is still a notoriously difficult problem [14, 57–59], to achieve this goal we consider all overrepresented motifs returned by DREME [36] in each extended binding peak datasets to maximally include possible true motifs. As shown in Figure 3B, depending on the size and quality of the datasets, a varying number (0 ~ 231) of motifs were found in each dataset. Particularly, in a total of six datasets that generally contain fewer binding peaks and are of low quality (26, 26, 28, 28, 70 and 5,188 sequences, Figure 3B), none or only a single motif could be identified. As no motif pairs can be formed in these datasets, they did not contribute to the final CRE and CRM predictions. In other words, they were filtered out by the motif-finder. On the other hand, putative CREs were found in the vast majority (99.98%) of the 439,886 extended binding peaks in the remaining 162 datasets, indicating that they were highly enriched with motifs. In this sense, the motif finding step servers as a quality control to filter out low quality datasets without the need of human involvement, conferring additional robustness to the algorithm. The returned motifs from the 162 datasets for 56 TFs (no TF was eliminated by discarding the six datasets) generally have high information contents (Figure 3C). Importantly, the known motifs of the ChIP-ed TFs were found by DREME for 99 of the 162 datasets, and were generally ranked high by the program, although they were usually not the top hit of DREME (Figure 3D), suggesting that it is necessary to consider a sufficient number of returned motifs to include the true ones. Moreover, when the datasets of different TFs have significant overlaps, we can identify all the motifs of the ChIP-ed TFs in all the overlapping datasets. For instance, the dataset GSM511084 for TF KR is significantly overlap with the dataset GSM511081 for TF HB, and motifs highly similar to the known binding sites of KR and HB were found in both the datasets (Figure 5B). Overall, we identified a total of 17,890 putative motifs corresponding to 35,359,819 putative CREs in the 162 datasets. These 35,359,819 putative CREs contain 275,857,398 bp which are 1.6 times of the genome, but only cover 30.9% (52,078,901 bp) of the genome, indicating that some of them still overlap with one another. At least one putative CRE was found in 1,061 (79.8%) of the 1,330 known CRMs in the sequences (Table 1). The failure to find CREs in the remaining 269 known CRMs in the datasets could be due to the fact that the CREs in these CRMs were not enriched in the datasets. Nonetheless, these results strongly suggest that in addition to the CREs of the ChIP-ed TFs, CREs of cooperative TFs, and thus at least partial CRMs are highly enriched in the extended binding peaks. This conclusion is in agreement with an early study based on 38 ChIP datasets in *D. melanogaster* [42], and also is supported by two recent studies using human datasets [56, 60].

**Table 1:** **Summary of the predictions of CREs and CRMs in the** ***D. melanogaster*** **genome at the major steps of the algorithm**

Steps	Motifs		CPs		CPCs		CRMC	CRM	Known CRMs	
	Number	Percentage	Number	Percentage	Number	Percentage			Number	Percentage
Motif findings	17890	NA	1,308,592	N/A	N/A	N/A	N/A	N/A	1,061	79.77%
CP finding	1589	8.88%	4,891	0.37%	N/A	N/A	N/A	N/A	1,041	98.11%
CPC finding	1376	86.60%	2,842	58.11%	951	N/A	N/A	N/A	1,036	99.52%
CRMC finding	1316	95.64%	2,807	98.77%	937	98.53%	815	N/A	1,036	100.00%
CRM finding	N/A	N/A	N/A	N/A	N/A	N/A	746	115,932	947	91.41%
Overall percentage		7.36%		0.21%		98.53%				77.89%

Each percentage is calculated based on the immediate previous step, except for the overall percentages which are based on the relevant initial step.

### Prediction of CRMs by iteratively enriching repeatedly used motif combinatorial patterns

**Figure 6:**
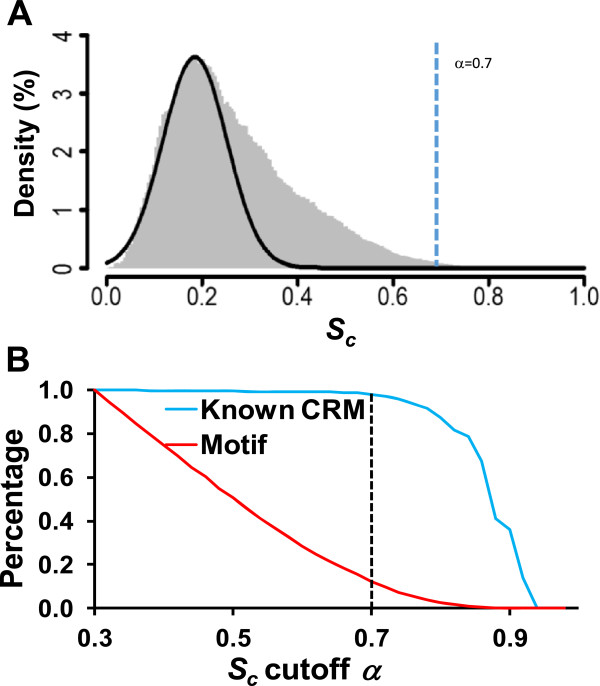
**Setting S**_**C**_ **cutoff α. A**. Distribution of co-occurring scores *S*_*c*_ of the motif pairs found in the 16 datasets. The curve is a fitting of the left portion of the distribution to a Gaussian distribution *N*(*μ* = 0.19, *σ* = 0.067). **B**. The remaining proportions of predicted motifs and known CRMs as functions of the *S*_*c*_ cutoff α. The vertical line indicates the position of the chosen cutoff α = 0.7 for selecting co-occurring motif pairs (CPs).

Clearly, as we used a rather loose stringency in motif finding to maximally include true motifs, there are inevitably a large number of spurious predictions in the 17,890 putative motifs identified in the datasets. Thus, our algorithm takes these 17,890 putative motifs as the input, and predicts CREs and CRMs by iteratively enriching repeatedly used motif combinatorial patterns though gradually filtering out spurious ones. Specifically, DePCRM first identifies highly co-occurring motif pairs (CPs) in each dataset by computing a co-occurring score (*S*_*c*_) (formula (2)) for each pair of putative motifs found in each dataset (Figures 2C). As shown in Figure 6A, the distribution of *S*_*c*_ is strongly skewed toward right, indicating that there are multiple components of the *S*_*c*_ values. The left low-scoring component can be well fitted to a Gaussian distribution with a mean and standard deviation 0.19 and 0.0043, respectively. The motif pairs accounting for this component are more likely to co-occur by chance, and thus they are likely spurious motif pairs. On the other hand, the right high-scoring portion of the distribution is more likely to attribute to true cooperative motif pairs. To find the *S*_*c*_ cutoff α by which a maximal number of motif pairs occurring by chance are filtered out while a maximal number of possible true motif pairs are kept, we plotted the proportion of the motif pairs with a *S*_*c*_ *≥* α as a function of α. As shown in Figures 5A and B, when α = 0.7, 1,303,701(1,303,701/1,308,592 = 99.6%) motif pairs and 16,301 motifs (16,301/17,890 = 91.1%) were filtered out, while putative CREs in only 20 (1.8%) the known 1,061 CRMs containing predicted CREs were completely left out. Thus we selected the motif pairs with *S*_*c*_ *≥* α = 0.7 as CPs for further analysis, thereby discarding the vast majority of presumably randomly occurring motif pairs (99.63%) and motifs (91.12%). This results in 4,891(4,891/1,308,592 = 0.4%) CPs containing 1,589 (1,589/17,890 = 8.9%) motifs (Table 1) for further analysis, which are presumably enriched for true motif pairs and motifs.

**Figure 7:**
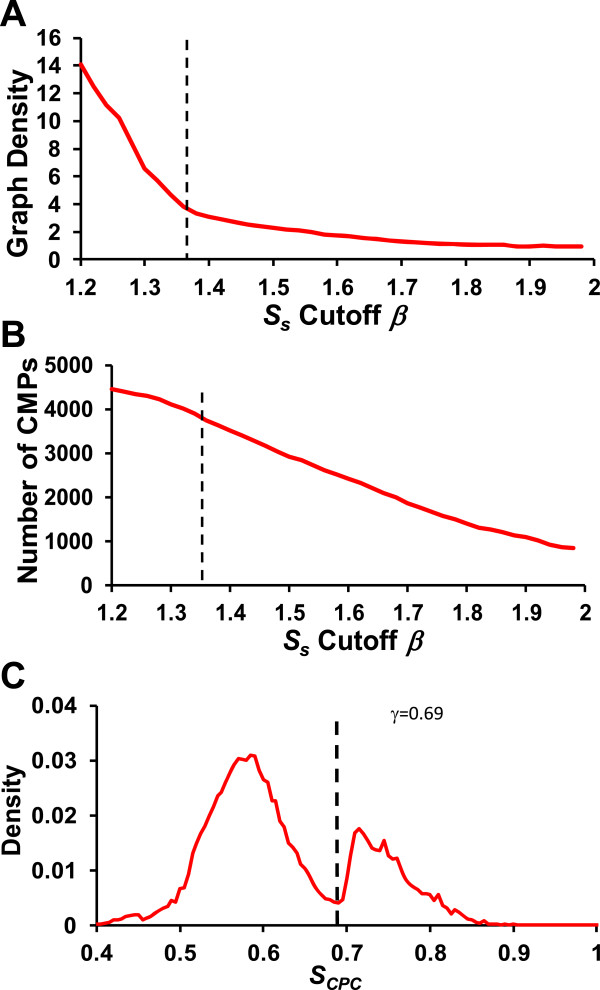
**Setting S**_**S**_ **cutoff β and S**_**CPC**_ **cutoff γ. A**. The density of the CP similarity graph drops rapidly with the increase in the *S*_*s*_ cutoff *β*, but the trend of decrease slows down around *β* =1.36. **B**. The number of CRM in the graph also starts to drop rapidly around *β* = 1.36. Thus we set *β* =1.36 for construing the final CP similarity graph. **C**. The distribution of CPC co-occurring scores *S*_*CPC*_ are well separated into a low-scoring component and a high-scoring component. The vertical line indicates the *S*_*CPC*_ cutoff *γ* =0.69 at the deepest valley between the two peaks, for constructing the CPC co-occurring graph.

To further enrich true motif pairs and motifs, the algorithm identifies repeatedly used CPs by clustering highly similar CPs in different datasets. To this end, we computed a similarity scores *S*_s_ (formula (3)) for each pair of CPs, each from two different datasets; and then constructed a CP similarity graph based on an *S*_s_ cutoff value *β* (Figure 2C). As shown in Figure 7A, with the increase in *β*, the density of the graph drops rapidly, but the dropping starts slowing down around *β* =1.36; meanwhile the number of nodes (CPs) in the graph starts decreasing rapidly around *β* =1.36 (Figure 7B). Thus, we set *β* =1.36 to construct the CP similarity graph (Methods). Applying the Markov chain clustering (MCL) algorithm [61] to the graph (Figure 2D) resulted in 951 CP clusters (CPCs) containing 2,842 (2,842/4,891 = 58.1%) CPs and 1,376 (1,376/1,589 = 86.6%) motifs (Table 1). Thus we further filtered out 2,049 (2,049/4,891 = 41.9%) CPs and 213 (213/1,589 = 13.4%) putative motifs.

Next, to identify larger repeatedly used motif patterns, we computed a co-occurring score *S*_*CPC*_ (formula (5)) for each pair of CPCs across the datasets in which both the CPCs have motifs. Interestingly, as shown in Figure 7C, the *S*_*CPC*_ scores display a well-separated bimodal distribution, and the low-scoring peak is likely mainly due to random motif patterns, while the high-scoring one is more likely attributable to truly cooperative motifs, thus we considered CPC pairs with an *S*_*CPC*_ ≥ *γ* =0.69 (at the valley between the two peaks) for further analysis. Applying the MCL algorithm to the resulting CPC co-occurring graph (Figure 2D and E, Methods), gave rise to 815 CRM components (CRMCs) containing 937(937/951 = 98.5%) CPCs, 2,807(2,807/2,842 = 98.8%) CPs and 1,316 (1,316/1,376 = 95.6%) motifs (Table 1). The compositions and structures of these 815 CRMCs are shown in Additional file 6: Figure S3, each containing 1 ~ 9 CPCs. Overall, 16,574 (92.6%) of the original 17,890 input motifs were filtered out by the algorithm (Table 1), suggesting that at least the vast majority (92.6%) of the putative motifs found in the datasets are spurious predictions.

As expected, some of the resulting 1,316 motifs found in different datasets are highly similar and often overlap with one another as demonstrated by the examples shown in Figure 5B. They are likely recognized by the same TFs or closely related ones, thus need to be combined into non-redundant and unique ones. To this end, we iteratively clustered the final 1,316 motifs based on their similarities (Methods), resulting in 184 clusters. We consider each cluster as a unique motif and refer to it as a Umotif, each containing 1 or 2 ~ 108 highly similar motifs and 255 ~ 88,702 CREs (Additional file 7: Figure S4, Additional file 8: Table S3). When compared with the known motifs in multiple built-in databases including DMMPMM, iDMMPMM, flyreg and fly factor survey using TOMTOM [62–65], 111 (60.3%) of the Umotifs are highly similar to known motifs in *D. melanogaster* at *p* < 0.001 (Additional file 8: Table S3), strongly suggesting that they are likely to be true motifs. As shown in Additional file 9: Figure S9, a p-value cutoff of 0.001 is sufficient to identify highly similar motifs. More examples of such Umotifs, their constituent motifs and the known motifs hit are shown in Additional file 10: Figures S5A and 5B. The rest 73 Umotifs that does not resemble any known motif might be novel ones. Examples of such Umotifs, their constituent motifs are shown in Additional file 10: Figures S5C and 5D. Furthermore, 106 (29.4%), 203 (56.2%) and 269 (74.5%) of 381 possibly redundant motifs found in the earlier study [42] were recovered by the Umotifs with a p-value cutoff of 0.001, 0.005 and 0.01, respectively. We replaced the motifs in the CRMCs with the Umotifs that they belong to, and each of the CRMCs is represented by their constituent Umotifs. Some CRMCs contain the same combination of Umotifs, thus we merged them in a unique one, resulting in 746 CRMCs.

### Genome-wide predictions of CREs and CRMs in *D. melanogaster*

**Figure 8:**
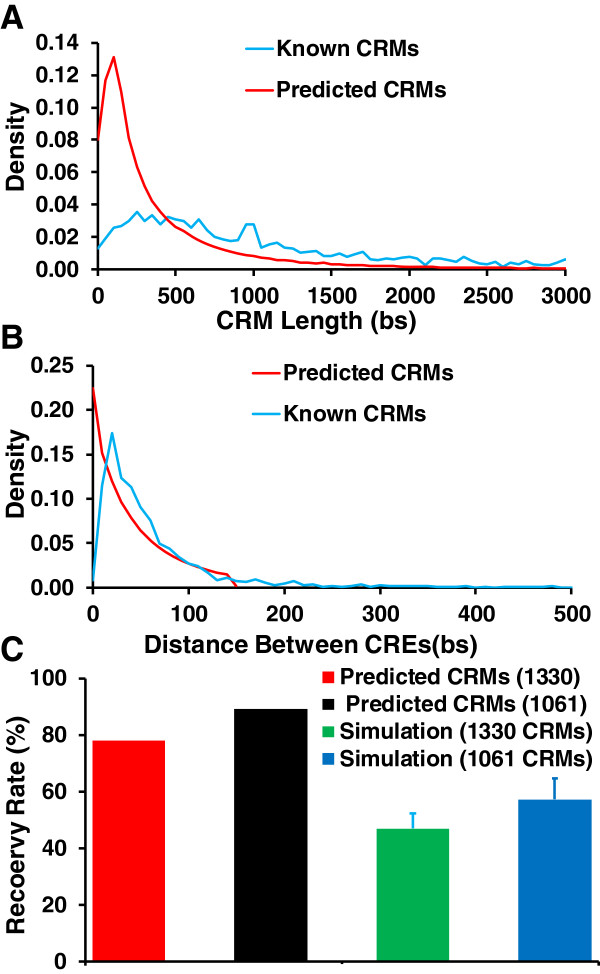
**Summary of the predicted CRMs. A**. Distribution of the lengths of the known and predicted CRMs. **B**. Distribution of the distances (bp) between two adjacent CREs in the known and predicted CRMs. **C**. Recovery rates of the known CRMs in the datasets (1330) and the known CRMs containing a predicted CRE (1061) by the predicted CRMs and the corresponding same number and length sequences randomly selected from NCRs.

Projecting the CREs in these 746 CRMCs back to the *D. melanogaster* genome (Methods) resulted in a total of 1,108,018 non-overlapping CREs with an average of 8.2 ± 2.8 bp, with 53,785 (4.9%) of which being entirely located in CDRs. These 1,108,018 CREs cover 9,045,115 bp (5.4%) genome sequence, of which 8,583,816 bp (94.9%) are in NCRs, consisting of 6.4% of NCRs; the remaining 461,299 bp (5.1%) are in CDRs, consisting of 1.3% of CDRs (Figure 4 and Additional file 3: Table S2). By connecting these putative CREs (Methods), we predicted a total of 115,932 non-overlapping CRMs, 71,817 (61.9%) of which are entirely located in NCRs, and the remaining 44115 (38.1%) contain CDRs. These 115,932 CRMs cover 49,796,159 bp (29.5%) genome sequence, 46,880,944 bp (94.1%) of which are in NCRs, consisting 34.9% of NCRs; the remaining 2,925,215 bp (5.9%) are in CDRs, consisting of 8.4% of CDRs (Figure 4 and Additional file 3: Table S2). These putative CRMs tend to have shorter lengths than those of the known CRMs (Figure 8A). Furthermore, the putative CRMs harbor 2 to 146 with a median of 7 CREs, and the distances between adjacent two putative CREs are largely similar to those in known CRMs, except that a small portion of the putative CRMs tends to have a short distance between adjacent two putative CREs (Figure 8B). These results suggest that we might have missed certain CREs in the predicted CRMs, particularly at the two ends, presumably due to insufficient information in the limited number of available ChIP datasets used in this study. In other words, some of our predictions might consist of only a part of real CRMs with possible missing CREs at the two ends of the CRM. Clearly, in order to make more accurate and complete predictions, more and highly diverse ChIP datasets are needed.

To evaluate the sensitivity of our predicted CRMs, we first computed the recovery rate by the predicted CRMs of the 1,330 known CRMs contained in the datasets. We consider a known CRM is recovered if it overlaps with a predicted CRM by at least half of its length. Remarkably, 1,036 (77.9%) of the 1,330 known CRMs were recovered by the 115,932 putative CRMs (Table 1). By contrast, when the same number and length sequences were randomly selected from the genome region covered by the datasets, only 46.9 ± 5.5% (*n* = 50) (Figure 8C) of the 1,330 known CRMs could be recovered. The recovery rate for the 1,061 known CRMs, in which at least a putative CRE was found, was even higher (947/1,061 = 89.3%). By contrast, when the same number and length sequences were randomly selected from the genome region covered by the dataset, only 57.2 ± 7.6% (*n* = 50) (Figure 8C) of the known CRMs were recovered. Hence, our algorithm has achieved rather a high recovery rate or sensitivity of CRM predictions, in particular when a putative CRE could be identified in them, even using just the limited number of datasets for only 56 TFs. Importantly, some of known CREs in these recovered CRMs overlap with our predicted CREs. For example, CRM(3R:21859748.. 21862775) containing Umotif 34 recovers a known CRM of gene *e*(*spl*); and a putative CRE of Umotif 34 overlaps with the known CRE of TF DA in the CRM, while Umotif 34 is highly similar to the known motif of DA (Additional file 11: Figure S6A). Furthermore, CRM (2 L: 15731775..15732968) containing Umotifs 106 and 114 recovers the known CRM of gene *cycE*; moreover, Umotifs 106 and 114 are highly similar to the known motifs of HTH and KNI which also have CREs located in the recovered CRM, respectively (Additional file 11: Figure S6B and S6C). In addition, many of our novel predictions also have strong experimental data supports thus are likely to be authentic. For example, our predicted CRMs 3R:8896195..8898063 , 3R: 12636031..12636729 and 2R: 5984055..5984519 share Umotifs 3 and 14, and they recover the known CRMs of genes *abd-A*, *jun-realted antigen* (*jra*) and *single-minded* (*sim*). It has been shown that these three genes are involved in nervous system development [66–68], and thus are likely to be coregulated. Consistent with this, we identified CREs of Umotifs 3 and 14 in the regulatory regions of these genes. Interestingly, Umotifs 3 and 14 are highly similar to the known motifs of hormone receptor 51 (HR51) and ladybird early (LBE), receptively (Additional file 11: Figures S6D and S6E), and it has been reported that HR51 and LB regulate neurogenesis [69, 70]. Thus HR5 and LB might carry out their functions by binding to the putative CREs of Umotifs 3 and 14. Furthermore, we have predicted a CRM 2R: 16831599..16832019 overlaps with the first intron of gene *actin57B* (Additional file 12: Figure S7) containing Umotif 27 and 23, which are highly similar to the known motifs of TFs myocyte enhancer factor 2 (MEF2) and chorion factor 2 (CF2), respectively (Additional file 11: Figures S6F and S6G). It has been shown that these two TFs cooperatively regulate Actin57B by binding to its promoter region [71]. Thus MET2 and CF2 might also regulate *actin57B* through binding to the putative CRES of Umotifs 27 and 23 located in its first intron (Additional file 12: Figure S7). Therefore, our predicted CREs and CRMs can help biologists identify potential enhancers for genes of interest.

### The predicted CRMs as well as CREs in a CRM as a whole are more conserved than randomly selected sequences

**Figure 9:**
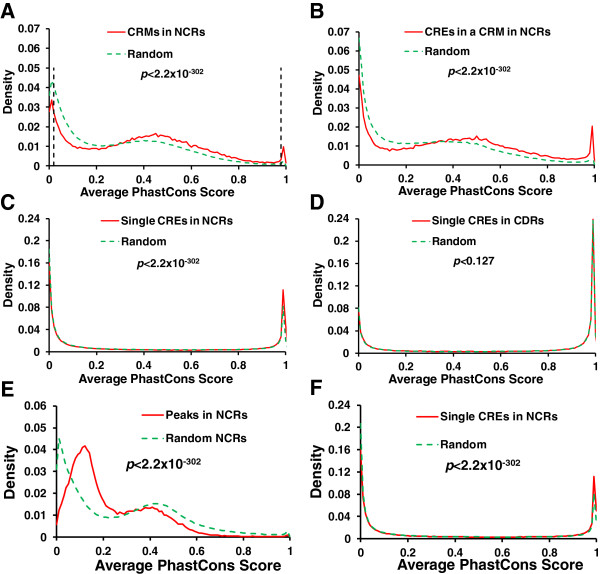
**Conservation analysis of the CRMs. A**. Distribution of average phastCons scores of the predicted CRMs in NCRs and of the same number and length sequences randomly selected from NCRs. The vertical dashed lines indicate the PhastCons score cutoffs for highly conserved (≥0.98) and non-conserved (≤0.02) CRMs. **B**. Distribution of average phastCons scores of all putative CREs in a predicted CRMs in NCRs and of the same number and length sequences randomly selected from NCRs. **C**. Distribution of average phastCons scores of single predicted CREs in NCRs and of the same number and length sequences randomly selected from NCRs. **D**. Distribution of average phastCons scores of single predicted CREs in CDRs and of the same number and length sequences randomly selected from CDRs. **E**. Distribution of average phastCons scores of the non-redundant original binding peaks in NCRs and of the same number and length sequences randomly selected from NCRs. **F**. Distribution of average phastCons scores of single predicted CREs in the original binding peaks in NCRs and of the same number and length sequences randomly selected from the original binding peaks in NCRs.

As functional sequences tend to be more conserved than non-functional ones, to further evaluate our predicted CRMs and CREs, we first compared the average phastCons conservation scores [72] of the nucleotides in each of the putative 71,817 CRMs entirely located in NCRs with those of the same number and length sequence randomly selected from NCRs. The phastCons score is computed as the posterior probability for a nucleotide to be conserved given a multiple alignment of genomes and their phylogenetic tree [72]. As shown in Figure 9A, although the average phastCons scores of both the predicted CRMs in NCRs and the randomly selected sequences have tri-modal distributions, they are significantly different (*p* < 2.2×10^-302^, Kolmogorov–Smirnov test). Specifically, the right peak with very low phastCons scores, which reflects highly conserved sequences [72] is much larger for the former than for the latter, and the opposite is true for the left peak with very high phastCons score, which reflects highly non-conserved sequences [72]. Moreover, the middle peak with intermediate phastCons scores, which reflects neutral to moderately conserved sequences [72], shifts about 0.04 to right for the former relative to that for the latter. Thus the nucleotides in the predicted CRMs in NCRs tend to be more conserved than those in the randomly selected sequences. As the spacing sequences between CREs in a CRM may not necessarily be functional and thus conserved, we next compared average phastCons scores of putative CREs in each of the 71,817 predicted CRMs in CDRs with those of the same number and length sequences randomly selected from NCRs. As shown in Figure 9B, average phastCons scores of CREs in a CRM and randomly selected sequences from NCRs also show tri-modal distributions, however again, they are significantly different (*p* <2.2×10^-302^, Kolmogorov–Smirnov test) in the similar way as for those of the full length putative CRMs and the corresponding randomly selected sequences (Figure 9A). However, there are subtle differences between the two cases: compared to the difference between the peaks for the putative CRMs and the randomly selected sequence (Figure 9A), the right peak for the putative CREs is much larger than that of the randomly selected sequences (Figure 9B), and the middle peak for the putative CREs shift more (0.15 vs. 0.04 unit) to right relative to that of the randomly selected sequences (Figure 9B). Hence, putative CREs in a CRM as a whole are much more conserved than the randomly selected NCRs, and also more conserved than spacer sequences in the putative CRMs. We further compared average phastCons scores of nucleotides in single CREs in the 71,817 predicted CRMs in CDRs and in the 53,785 predicted CRMs in CDRs with those of the same number and length sequences randomly selected from NCRs and CDRs, respectively. As shown in Figure 9C and D, the average phastCons scores of single putative CREs in both NCRs and CDRs and those of the corresponding randomly selected short *k*-mer sequences all show well separated bi-modal distributions, with each peak located near the two extremes (0 and 1) of phastCons scores. This result indicates that nucleotides in single putative CREs in both NCRs and CDRs and their corresponding randomly selected short *k*-mers all tend to have either a very low (near zero) or a very high (near 1) average phastCons score, implying that the nucleotides in short sequences tend to be simultaneously highly conserved or non-conserved. This observation is consistent with the findings that the *D. melanogaster* genome is highly compact, and vast majority of its sequences are either negatively or positive selected, and thus are likely to be functional [73–79]. However, interestingly, there are striking differences between the predicted CREs in NCRs (Figure 9C) and those in CDRs (Figure 9D). First, the distribution for single putative CREs in NCRs is significantly different from that for the corresponding randomly selected sequences (*p* < 2.2×10^-302^, Kolmogorov–Smirnov test), as the right peak of the former is slightly larger than that of the latter (Figure 9C), indicating that a small fraction of single predicted CREs in NCRs are more conserved than the randomly selected short *k*-mers. By contrast, the distributions for single putative CREs in CDRs and the corresponding randomly selected short *k*-mers are not significantly different (p < 0.127, Kolmogorov–Smirnov, Figure 9D), indicating that single putative CREs in NCRs are not more conserved than the randomly selected short *k*-mers. Second, the right peaks for single predicted CREs in NCRs and the randomly selected short *k*-mers are slightly smaller than their own left peaks (Figure 9C), indicating that there are slightly fewer conserved short sequences than non-conserved ones in NCRs. By contrast, the right peaks for single putative CREs in CDRs and the randomly selected short *k*-mers are much larger than their own left peaks (Figure 9D), indicating that there are much more conserved short sequences than non-conserved ones in CDRs, which is expected as most CDRs are highly conserved. Third, the right peaks for single putative CREs in NCRs and the corresponding randomly selected *k*-mers are much smaller than those of single putative CREs in CDRs and the corresponding randomly selected short *k*-mers, and the opposites are true for the left peaks (Figure 9C and D), indicating that short sequences in CDRs are more conserved than those in NCRs as expected. Finally, to see the extent to which the original binding peaks (without length extension) in the datasets were enriched for CRMs and CREs, we computed average phastCons scores of the non-redundant original binding peaks and the CREs contained as well as of the same number and length sequences randomly selected from NCRs and NCRs in the binding peaks, respectively. As shown in Figure 9E, the distribution of average phastCons scores of non-redundant original binding peaks was quite different from that of putative CRMs. In particular, the peak at the score = 1 in the latter distribution was almost missing in the former distribution. Moreover, the original binding peaks with an average phastCons score >0.32 even tended to be less conserved than randomly selected NCRs, and the opposite was true for the putative CRMs, indicating that the predicted CRMs contains more conserved sequences than do the original binding peaks. Furthermore, the distribution difference between average phastCons scores of CREs predicted in the original binding peaks and those of randomly selected NCRs with the same lengths is similar to that between average phastCons scores of CREs and those of the randomly selected NCRs (Figure 9F). Thus our predicted CREs in extended binding peaks as a whole are of similar quality to the predicted CREs in the original binding peaks. In summary, although only a small fraction of the single predicted CREs in NCRs are more conserved than the randomly selected short *k-*mers, predicted CREs in a putative CRM as a whole and predicted CRMs are significantly more conserved than the corresponding randomly selected sequences, thus they are highly likely to be functional.

### Highly conserved and non-conserved CRMs regulate distinct classes of genes

To further evaluate our predicted CRMs, we examined whether or not the highly conserved predicted CRMs (with an average phastCons score ≥0.98) and highly non-conserved predicted CRMs (with an average phastCons score ≤0.02) (Figure 9A) have distinct regulatory functions. To this end, we assigned each of the predicted CRMs a target gene whose transcription start site has the shortest distance to the predicted CRM. Thus a predicted CRM can only be assigned to a gene, while a gene can have multiple assigned putative regulating CRMs. A total of 763 and 2,319 genes are predicted as targets of the highly conserved and highly non-conserved putative CRMs, of which 601 and 2,053 have gene ontology (GO) annotations, respectively. As shown in Additional file 13: Table S4, 134 (22.3%) the putative target genes of the 601 highly conserved putative CRMs are clustered into 11 functional groups using the DAVID program [80] with an enrichment score ≥1.5 and p < 0.01 (hypergeometric test with Benjamini correction). Intriguingly, these genes are enriched for developmental functions (8 groups), neurological functions (1 group), motility (1 group) and transcriptional regulations (1 group). On the other hand, 481(23.4%) putative target genes of the 2,053 highly non-conserved putative CRMs are clustered into 10 functional groups with an enrichment score ≥1.5 and p < 0.01, In contrast to the putative target genes of highly conserved putative CRMs, these genes are enriched for plasma membrane functions (6 groups), metabolism (2 groups), and chemical sensory perception (2 groups) (Additional file 14: Table S5). Thus, the highly conserved putative CRMs and highly non-conserved putative CRMs do regulate distinct groups of genes. The results are in excellent agreement with the fact that highly conserved CRMs are mainly involved in embryonic development in both insects [81, 82] and vertebrates [83], while CRMs for genes with other functions in particular those related to environmental adaptations evolve extremely fast [84], strongly suggesting that both the highly conserved putative and non-conserved putative CRMs are likely to be functional. The predicted CREs, Umotifs, CRMs, average phastCons scores and putative target genes are stored in a searchable relational database PCRMs (http://bioinfo.uncc.edu/mniu/pcrms/www/) for public use. The query results and relevant knowledge are displayed using the NCBI graphical sequence viewer. Currently, the algorithm was implemented in Perl, and the scripts are available on the PCRMs website.

## Discussion

The recent development of various ChIP-seq, DNase-seq and FAIRE-seq techniques for locating bind regions of specific TFs, chromatin marks, free nucleosome regions, has provided an unprecedented opportunity for deciphering all *cis*-regulatory sequences in eukaryotic genomes. These techniques and resulting datasets reveal similar or quite different aspects of *cis*-regulatory sequences, and have their pros and cons. On one hand, a single epigenomic dataset resulted from DNase-seq, FAIRE-seq, or enhancer mark such as H3K27ac ChIP-seq provide information of the locations of all functional CRMs in a cell or tissue type, thus these techniques could be less expensive. However, it is very difficult to predict novel CREs of specific TFs from such an epigenetic dataset, since it lumps the potential CREs for all TFs active in the cell or tissue type, and TF information is usually unavailable. Due to the lack of CRE locations, it is also difficult to predict CRMs at single nucleotide resolution using epigenetic datasets. On the other hand, a TF ChIP-seq dataset is highly enriched for the CREs of the ChIP-ed TF and of its cooperators, thus all these CREs can be potentially identified at single nucleotide resolution in a cell or tissue type. However, as a TF ChIP-seq dataset only contains location information of CREs of the ChIP-ed TF, a certain number of TFs that are potentially active in the cell or tissue type need to be analyzed to identify all the CREs and CRMs. Nevertheless, to fully understand the *cis*-regulatory genome and also for a wide spectrum of applications, it is necessary to real the exact locations of all CREs and CRMs in the genome. ChIP-seq datasets for various TF in different cell and tissue types can be the key to the goal.

However, precise identification of CREs in the binding peaks from ChIP experiments is still a challenging computational problem [59]. Efforts have been made to narrow down the binding peaks through improving experimental procedures [85], thereby facilitating the identification of CREs. On the other hand, once the binding peak summits of a TF are identified, information about the CREs of its cooperative TFs around the summits can provide a good opportunity to identify the relevant CRMs. With the accumulation of a large number of ChIP datasets in many important metazoans and plants, it is tantalizing to predict CRMs around CREs of the ChIP-ed TFs by integrating information in a large number of ChIP datasets in an organism. In this study, we have explored this idea and developed a novel algorithm DePCRM for such a purpose. The algorithm is largely based on the fact that similar TF combinatorial patterns are often repeatedly used to regulate multiple similar or different regulons in different cell types, tissues, developmental stages or physiologically conditions. As the number of possible combinations of TFs is extremely large, DePCRM identifies possible real motif combinatorial patterns in a sufficiently large number of ChIP datasets through iteratively filtering out randomly occurring spurious motifs, thereby effectively reducing the searching space in each step (Table 1). Clearly, in order for the algorithm to make reasonable predictions, the ChIP datasets have to be sufficiently large and diverse, so that they are likely to include datasets for cooperative TFs in different cell types, tissues, developmental stages and physiological conditions.

Using the currently available 168 ChIP datasets for 56 TFs in *D. melanogaster*, the algorithm was able to recover 77.9% of the known CRMs in the datasets and even 89.3% known CRMs in which a putative CRE could be identified (Table 1). Thus our algorithm has achieved rather high prediction sensitivity even only using these limited 168 datasets, in particular when a putative CRE can be located in the CRMs by a motif finding tool. Although we cannot rigorously evaluate the prediction specificity of the algorithm due to the limited knowledge of CRMs in the genome, it should not be too low for the following reasons. First, the chance for such high recovery rate of known CRMs to happen by chance is virtually impossible as indicated by our simulation studies (Figure 8C). Second, our predicted CRMs as well as CREs in a CRM as a whole are more conserved than the corresponding randomly selected sequences (Figure 9A and B). Third, the highly conserved predicted CRMs tend to be located in the close neighborhoods of genes involved in embryonic development (Additional file 13: Table S4), which is consistent with the existing knowledge [81–83]. Fourth, the highly non-conserved predicted CRMs tend to be located in the close neighborhoods of genes involved in neural transmission, chemical sensation and metabolism, which is also in excellent agreement with the observations that gene regulatory networks for genes involved in responses to environmental factors tend to evolve very rapidly through rewiring by degrading existing CREs (death), or gaining new CREs (birth), a process called CRE turnover [84, 86]. This form of genetic changes plays a more pivotal role in functional evolution of organisms than previously thought [84, 87]. Therefore, both the conserved and non-conserved putative CRMs are highly likely to be functional.

As vast majority of known CRMs are located in NCRs, we did not attempt to predict CRMs that are entirely located in CDRs, thus, we only allow the extended binding peaks to include at most the adjacent exon (Methods). Nevertheless, 5.9% of our predicted CRMs at least partially include the first or last exon of genes. Although putative CREs in CDRs are more likely to be conserved than those in NCRs (Figure 9C and D), they are not more conserved than the randomly selected short *k*-mers in CDRs (Figure 9D). Therefore putative CREs in CDRs are not necessarily under a higher selection pressure than are the randomly selected short *k*-mers in CDRs. On the other hand, the other 94.1% of our predicted CRMs are entirely located in NCRs (Figure 4), and consist of 34.9% of all NCRs in the genome. Interestingly, it has been shown that there are more than three times as many functional NCRs as CDRs in the *D. melanogaster* genome, because these NCRs are under at least the same level of natural selection as CDRs [73, 75, 79]. In other words, more than 75% of NCRs in the genome are likely to be functional. In this regarding, we have predicted less than half of possible CRMs in the genome. Furthermore, our predicted CRMs are based on 746 combinatorial patterns (i.e., CRMCs) of 184 identified Umotifs. Since TFs of the same structural family tend to recognize highly similar motifs [88, 89], our predicted Umotif might correspond to multiple highly similar motifs of different TFs of the same structural family. Hence, we may have actually predicted more than 184 motifs for some of the 1,052 annotated TFs in the genomes, and many of them are likely novel motifs. However, our predicted motifs might be far away from covering all the annotated TFs as our predicted CRMs only cover 34.9% of NCRs, of which at least 75% are likely to have transcriptional regulatory functions [73, 75, 79].

Nonetheless, our results demonstrate that even these limited 168 datasets for just 56 TFs can result in highly meaningful predictions of CRMs and CREs genome-wide. In other words, these datasets contain sufficient information for repeatedly used motif patterns as indicated by the significant overlaps of their binding peaks (Figure 4 and Additional file 5: Figure S2). On the other hand, because these datasets were not generated by random efforts of the community, rather, they are likely biased to well-studied cooperative TFs, and their CRMs are relatively well documented in the literature. Therefore, if the datasets were generated by random efforts and the known CRMs were characterized by uncorrelated efforts, then we might need a much larger number of datasets to achieve the similar prediction accuracy. Moreover, as indicated above, although we have achieved a rather high recovery rate (89.3%) of known CRMs with a putative CRE, more and diverse ChIP datasets are needed to further improve the predictions, in particular to predict all CRMs in the genome. Fortunately, with ChIP-seq techniques becoming routine and the progress of the ENCODE projects, more and more ChIP-seq datasets will be churned out for numerous and even all TFs encoded in the organisms. Thus our algorithm could be very useful for elucidating CRMs encoded any genome once a sufficient number of diverse ChIP-seq datasets become available in the organism.

Clearly, our predicted result is only a static map of CREs and CRMs encoded in the genome, and for many putative CREs in the predicted CRMs, we may not know their cognate TFs and functional states (active, poised or inactive) in specific cell types and tissues. However, once such a global CRMs map is available for an organism, it is relatively straightforward to infer the functional states to CRMs if epigenetic data in a certain cell type, tissue, developmental stage or physiological condition are available, such as ChIP-seq data for histone modification markers (e.g., mono-, bi- and tri-methylation at lysine 4 of histone 3 or H3K4m1, H3K4m2, K3K4m3, etc.) at active promoters, enhancers and silencers [7, 21, 90–93], and DNAse-seq data for nucleosome free regions [22, 24, 85, 94, 95]. In this sense, various epigenetic datasets in different cell and tissue types can complement with TF ChIP datasets and speed up the process of deciphering the entire cis-regulatory genome of an organism. Thus, a future development is to incorporate the epigenetic datasets, hereby predicting the functional states of all the predicted CRMs in a certain cell type, tissue, developmental stage or physiological condition [90–93]. Then, it is also possible to predict the molecular, cellular and organismal phenotypes based on the functional states of the CRMs and their variations among individuals and species, given the recent indication of the importance of CRMs in determining the phenotypes of organism [96–104].

## Conclusions

The exponentially increasing number of TF binding location data produced by the recent wide adaptation of chromatin immunoprecipitation coupled with microarray hybridization (ChIP-chip) or high-throughput sequencing (ChIP-seq) technologies has provided an unprecedented opportunity to identify CRMs and CREs in genomes. However, how to effectively mine the large volumes of ChIP data to identify CREs and CRMs is a challenging task. We have developed a novel graph-theoretic based algorithm DePCRM for genome-wide *de novo* predictions of CRMs and CREs using a large number of ChIP datasets. DePCRM predicts CRMs by identifying overrepresented combinatorial motif patterns in multiple ChIP datasets in an effective way. When applied to 168 ChIP datasets of 56 TFs from *D. melanogaster*, DePCRM identified 184 and 746 overrepresented motifs and their combinatorial patterns, respectively, and predicted a total of 115,932 CRMs in the genome. The predictions recover 77.9% of known CRMs in the datasets, 89.3% of known CRMs containing at least one predicted CRE. These putative CRMs and CREs as a whole in a CRM are more conserved than randomly selected sequences, thus, they are highly likely to be functional. Thus the algorithm can be used to predict CRMs and CREs in other eukaryotic genomes from which a sufficient number of diverse ChIP datasets are available. All the predicted CREs, motifs, CRMs, and their target genes are available at http://bioinfo.uncc.edu/mniu/pcrms/www/.

## Methods

### Datasets

We attempted to collect all possible ChIP-seq and ChIP-chip datasets from *D. melanogaster* available to us from three sources: the modENCODE project [46], the Berkeley drosophila transcription network project (BDTNP) [53] and literature. We used the binding peak summits in each dataset, provided in the original publications, as the data owners might have a better understanding of their datasets for background subtraction and normalization. We removed binding peaks that overlap with high occupancy target (HOT) regions [42, 43]. Because the typical lengths of known CRMs are 1,000-2,000 bp [54], we extended the binding peaks shorter than 3,000 bp to up to 3,000 bp by padding equal length of flanking genomic sequences to the two ends. If the extension on either end reaches to an adjacent exon, we only included up to the full length sequence of the exon as majority of CRMs are located in NCRs. We discarded the binding peaks longer than 5,000 bp as they generally have low quality score and consist of only a small portion in the datasets (Figure 3A). The remaining extended binding peaks in each dataset were used for motif finding. The known CREs and CRMs in *D. melanogaster* were downloaded from the REDfly database [54].

### Measurement of the overlap of binding peaks in two datasets

We quantify the overlapping level of binding peaks in two datasets *d*_*i*_ for TF *F*_*i*_ and *d*_*j*_ for TF *F*_*j*_, defined as,
1

where |*d*_*i*_| and |*d*_*j*_| are the number of binding peaks in *d*_*i*_ and *d*_*j*_, respectively, and *o*_*i*_(*d*_*i*_, *d*_*j*_) the number of sequences that have at least one pb overlap between the sequences in the two datasets.

### Finding motifs in binding peak datasets

Based on an initial evaluation of multiple motif-finding tools for large ChIP datasets, including seeder [30], Trawler [30, 31], ChIPMunk [32], HMS [33], CMF [34], STEME [35], DREME [36], DECOD [37], RSAT [38], and POSMO [39], we selected DREME to identify all possible motifs in each of the extended binding peak dataset for its computational efficiency and capability to return enough number of over-represented motifs in a dataset [36]. As DREME requires a negative dataset for more accurate predictions, we generated a random sequence set for each input dataset using a third order Markov chain model based on the transition probabilities of the sequences in the dataset. In addition, since it is highly unlikely that one can find a large number of high quality motifs in such a random dataset or in a low quality ChIP dataset, we also used DREME as a quality control measure to filter out low quality datasets in which no or only a single motif could be identified.

### The algorithm

Our DePCRM algorithm predicts CRMs through the following steps using the putative motifs as the input found in the modified binding peaks from all ChIP-seq and/or ChIP-chip datasets.

### Step 1 identify co-occurring motif pairs (CPs) in each dataset

For each pair of motifs *M*_*d*_(*i*) and *M*_*d*_(*j*), regardless of their distance found in the same dataset *d*, we compute a motif co-occurring score *S*_*c*_ defined as,
2

where |*M*_*d*_(*i*)| and |*M*_*d*_ (*j*)| are the number of binding peaks containing CREs of motifs *M*_*d*_(*i*) and *M*_*d*_ (*j*), respectively; and *o*(*M*_*d*_(*i*), *M*_*d*_(*j*)) the number of binding peaks containing CREs of both the motifs. We select motif pairs with a *S*_*c*_ ≥ α as co-occurring motif pairs (CPs) for further analysis (Figure 2B and C). The cutoff α is chosen such that the predicted motifs in known CRMs are minimally excluded (Figure 5A and B). If there are not enough known CRMs in the genome, a default α = 0.7 is used based on the data from REDfly (see Results).

### Step 2 compute similarity scores among all pairs of CPs in different datasets

For each pair of datasets *a* and *b*, we compute a similarity score *S*_*S*_ between each pair of CPs *P*[*M*_*a*_(*i*), *M*_*a*_(*j*)] from *a* and *P*[*M*_*b*_ (*m*), *M*_*b*_ (*n*)] from *b*, defined as,
3

where *Sim*(*M, N*) is the similarity score between motifs *M* and *N* using a metric called SPIC that we proposed previously considering both the frequency matrixes and position specific weight matrixes (PSWMs) of both the motifs [105–107]. We have shown that SPIC outperforms the existing metrics for measuring motif similarities [105–107]. Note that to compute S_*s*_ we first select the highest similarity among all the four possible motif pairs, and then sum it with the similarity of the remaining pair.

### Step 3 construct the CP similarity graph

We then construct a CP similarity graph using the CPs as the nodes, and connecting two CPs with an edge with their score *S*_*s*_ being the weight if and only if *S*_*s*_ is above a cutoff *β*. As edges are only allowed among CPs from different datasets, thus the resulting similarity graph is a multi-partied graph (Figure 2C). The value of *β* is chosen based on the relationship between the graph density as well as the number of nodes in the graph and different *β* values. The graph density is defined as:
4

where |*CP*| and *|E|* are the numbers of CPs and edges in the graph, respectively*.* We choose an *β* value such that the resulting graph is as spars as possible and has as many nodes/CPs as possible (Figure 7A and B).

### Step 4 cut the CP similarity graph into dense sub-graphs, CP clusters (CPCs)

We use the Markov Chain Clustering algorithm (MCL) [61] to cut the graph into dense sub-graphs, each corresponding to a cluster of repetitively occurring CPs across multiple datasets (Figure 2D). MCL iteratively computes random walks determined by a Markov chain by alternately executing two operations (expansion and inflation) on a stochastic matrix [61]. It ranks the identified dense sub-graphs according to their sizes in a descending order. It has been shown that MCL works very well in finding dense sub-graphs in very large weighted sparse graphs [61, 105, 106, 108–112]. We discard the clusters containing fewer than τ CPs (τ = 2 in this study, τthus we only discarded singleton CPs) (Figure 2D). Presumably, the remaining clusters contain highly similar CPs for certain two TFs. For example, cluster C1 (P1, P5, P8) in Figure 2D contains highly similar motifs (red and black ova) for two distinct TFs. For this reason we call these clusters CP clusters (CPCs) (Figure 2D).

### Step 5 compute a co-occurring score for each pair of CPCs

Let *C*_*i*_ and *C*_*j*_ be two CPCs, and *Ω*_*dk*_(*C*_*i*_, *C*_*j*_) be the set of the CPs in *C*_*i*_ and *C*_*j*_ from the same dataset *d*_*k*_. We define a co-occurring score between *C*_*i*_ and *C*_*j*_ as,
5

where *D* is the number of datasets in which CPs of both *C*_*i*_ and *C*_*j*_ occur, *P*_*s*_ and *P*_*t*_ two CPs from *C*_*i*_ and *C*_*j*_, respectively, o(*P*_*s*_,*P*_*t*_) the number of binding peaks where *P*_*s*_ and *P*_*t*_ co-occur, |*P*| the size of *P*, and *N*(Ω_*dk*_(*C*_*i*_*,C*_*j*_)) the number of unique comparisons among the CPs in Ω_*dk*_(*C*_*i*_*, C*_*j*_).

### Step 6 construct the CPC co-occurring graph

We construct a CPC co-occurring graph using each CPC as a node, and connecting two CPCs *C*_*i*_ and *C*_*j*_ by an edge with *S*_*CPC*_(*C*_*i*_, *C*_*j*_) being the weight if and only if *S*_*CPC*_(*C*_*t*_, *C*_*j*_) ≥ = *y* (Figure 2E). The cutoff *γ* is chosen based on the bimodal distribution of the *S*_*CPC*_ sores (Figure 7C).

### Step 7 cut the CPC co-occurring graph into dense subgraphs

We apply MCL to cut the CPC co-occurring graph into dense sub-graphs (Figure 2F). Each of these sub-graphs is assumed to correspond to a possible combination of their motifs to form a CRM based on the datasets used. For this reason, we refer to these CPC clusters as *CRM components* (CRMCs) (Figure 2E).

### Step 8 combine highly similar motifs in unique ones

Some motifs in the CRMCs may have overlapping CREs, and can be very similar to one another. It is highly likely that they consist of the same or similar CREs of the same TF or closely related ones. Thus we need to combine such highly similar and possibly redundant motifs into unique ones. To this end, we calculate the pairwise motif similarity of all the motifs in the CRMCs using the SPIC motif similarity metric [105–107]. We construct a motif similarity graph using the motifs as nodes, and connecting two nodes by an edge with the similarity being the weight if and only if the similarity of the corresponding motifs is greater than 0.7. We identify high density subgraphs in the graph using MCL. For each subgraph, we extend each CRE of each associated motif by padding 5 bp original genomic sequence at each of its two ends. We then identify the common motif in each set of the extended CREs using DREME. For the resulting motifs with more than 50% CRE overlapping and a similarity score more than 0.4, we repeat the above procedure until no two motifs meet the criteria. Each resulting motif has a similarity smaller than 0.4 and an overlapping rate lower than 0.5 with any other motifs. Thus we call each of them a unique motif or Umotif. Each motif in the identified CRMCs is then represented by its Umotif.

### Step 9 predict CRMs in the genome

We project CREs of all the CRMCs back to their locations in the genome. If the projected CREs overlap with one another, we merged them in a non-overlapping one. We then connect any two adjacent CREs if their distance is shorter than a preset value *δ* (*δ =* 150 bp in this study) according to the distribution of the distances between the CREs in known CRMs (Figure 8B) and the connection cannot span over an exon unless it contain a binding site. We predict as a CRM each segment of sequence connected by CREs of Umotifs in one or multiple CRMCs.

### Comparison of our algorithm with a naïve algorithm

Since CRMs are likely to be enriched in our extended peaks, a naïve method that randomly selects sequences from the extended peaks can recover true CRMs. To compare our algorithm with such a naïve method, we concatenated all the genome sequences that are covered by the extended binding peaks according to the order of the sequences on the chromosomes X, Y, 2, 3 and 4, and we connected the two ends of the concatenated sequence to form a circular DNA. For each of CRM predicted by our algorithm, we randomly selected a segment of sequence with the same length as the predicted CRM from the circular DNA. We repeated the process 50 times, and compared their averaged results to our predictions.

## Electronic supplementary material

Additional file 1: Table S1.: Summary of the 168 ChIP datasets we collected. (XLSX 17 KB)

Additional file 2: Figure S8.: Number of binding peaks in the 168 ChIP datasets we collected. Datasets are sorted in ascending order according to their sizes. (PDF 6 KB)

Additional file 3: Table S2.: Summary of the coverage of the datasets, predicted CRMs and CREs on the CDRs, NCRs and genome. (XLSX 9 KB)

Additional file 4: Figure S1.: An example of CRMs bound by TFs BCD, HB and KR from the REDfly database. The graph was shown using Gbrowser. (PDF 15 KB)

Additional file 5: Figure S2.: Hierarchical clustering of the 56 datasets for distinct TFs based on their pair-wise binding peak overlapping scores *S*_*o*_. The blow-up shows a cluster for cooperative TFs (see Results in the main text). (PDF 104 KB)

Additional file 6: Figure S3.: Structures of the 815 CRMCs. Each node in the graphs is a CPC, and each connected graph represents a CRMC. (PDF 87 KB)

Additional file 7: Figure S4.: Structures of the 184 Umotifs containing more than two motifs. Each node in the graphs is a putative motif, and each connected graph represents a Umotif. The logos are for the indicated Umotifs. (PDF 144 KB)

Additional file 8: Table S3.: Summary of the 184 Umotifs. (XLSX 20 KB)

Additional file 9: Figure S9.: Examples of Umotifs and their matched known motifs with a p-value around 0.001 using TOMTOM. (PDF 166 KB)

Additional file 10: Figure S5.: A. Umotif 72 and its four individual constituent motifs found in different datasets. Umotif 72 is similar to known motifs CG12287 and CG34395. B. Umotif 27 and its five individual constituent motifs. Umotif 27 is similar to known motif CG5249. C. Umotif 70 and its four individual constituent motifs found in different datasets. D. Umotif 93 and its two individual constituent motifs found in different datasets. (PDF 277 KB)

Additional file 11: Figure S6.: Examples of known CREs in the recovered known CRMs that overlap with our predicted CREs, their corresponding Umotifs are similar the known motifs. See main text for the details. (PDF 302 KB)

Additional file 12: Figure S7.: A putative CRM (shown in gray shadow) is located in the first intron of gene *act*57B. (PDF 180 KB)

Additional file 13: Table S4.: Enriched GO terms for the putative target genes of highly conserved putative CRMs. (XLSX 15 KB)

Additional file 14: Table S5.: Enriched GO terms for the putative target genes of highly non-conserved putative CRMs. (XLSX 20 KB)

## Abbreviations

CRE: *cis*-regulatory element
CRM: *cis*-regulatory module
bp: Base pairs
CDRs: Coding regions
ChIP: Chromatin immuneprecipitation
CPs: Co-occurring pairs
CPCs: Co-occurring pair clusters
CRMCs: CRM components
MCL: Markov chain clustering
NCRs: Non-coding regions
NGS: Next-generation sequencing
PSWMs: Position specific weight matrixes
TF: Transcription factor.
