# Supplementary figures and images for: De novo prediction of cis-regulatory elements and modules through integrative analysis of a large number of ChIP datasets
Source: BMC Genomics. 2014 Dec 2;15:1047. doi: 10.1186/1471-2164-15-1047 (PMC4265420; doi:10.1186/1471-2164-15-1047)

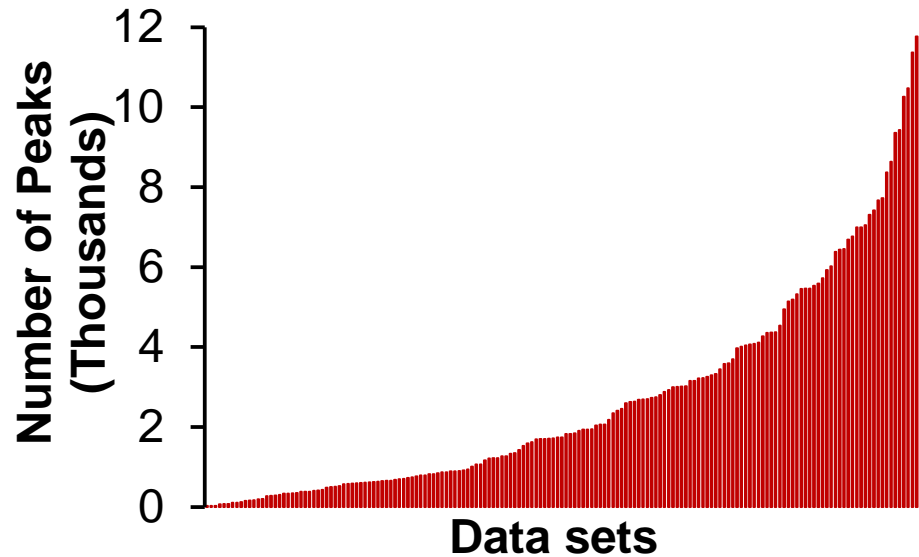

Supplement: Supplementary file 2 — Additional file 2: Figure S8.: Number of binding peaks in the 168 ChIP datasets we collected. Datasets are sorted in ascending order according to their sizes. (PDF 6 KB) [file 12864_2014_6723_MOESM2_ESM.pdf]

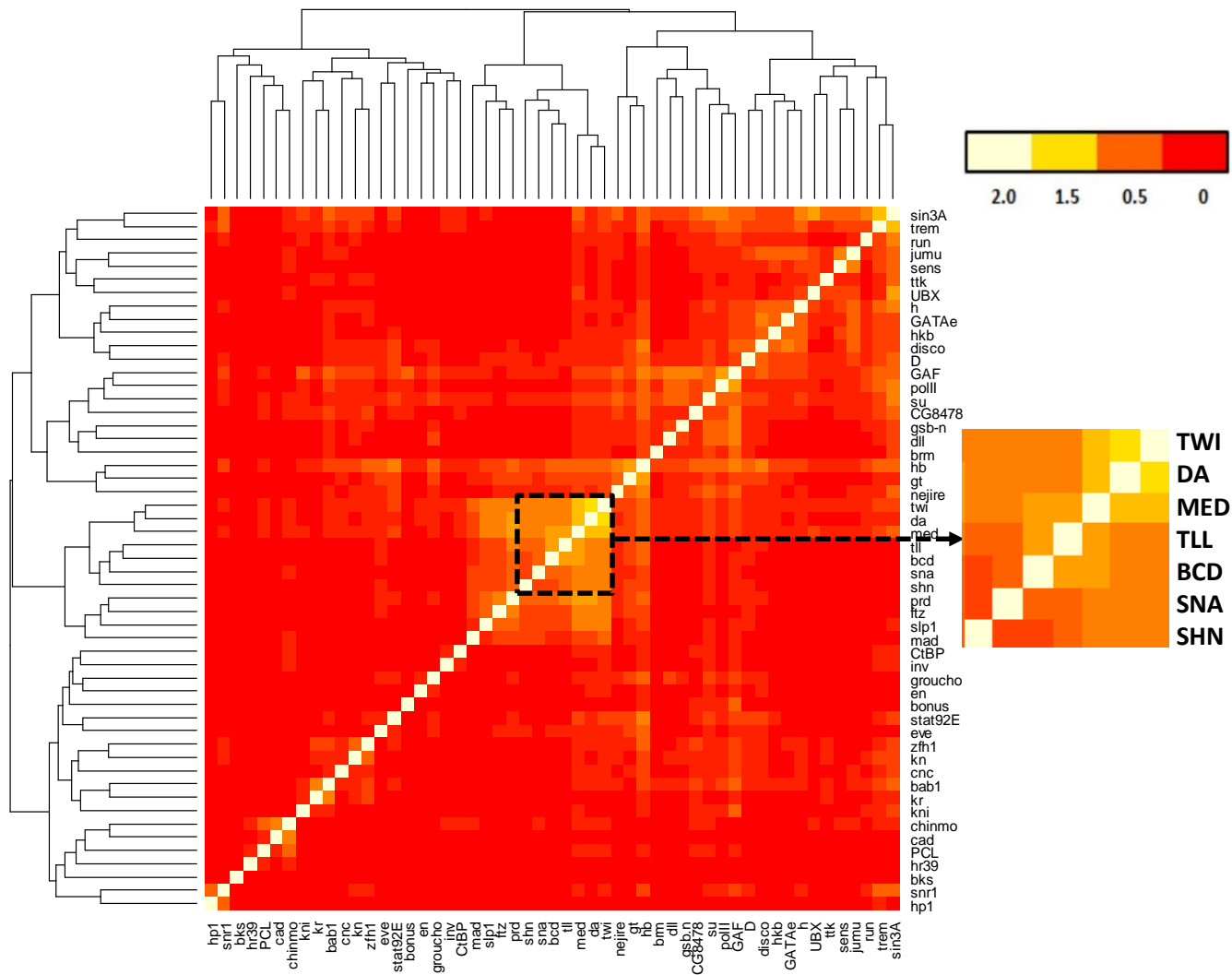

Supplement: Supplementary file 5 — Additional file 5: Figure S2.: Hierarchical clustering of the 56 datasets for distinct TFs based on their pair-wise binding peak overlapping scores S o. The blow-up shows a cluster for cooperative TFs (see Results in the main text). (PDF 104 KB) [file 12864_2014_6723_MOESM5_ESM.pdf]

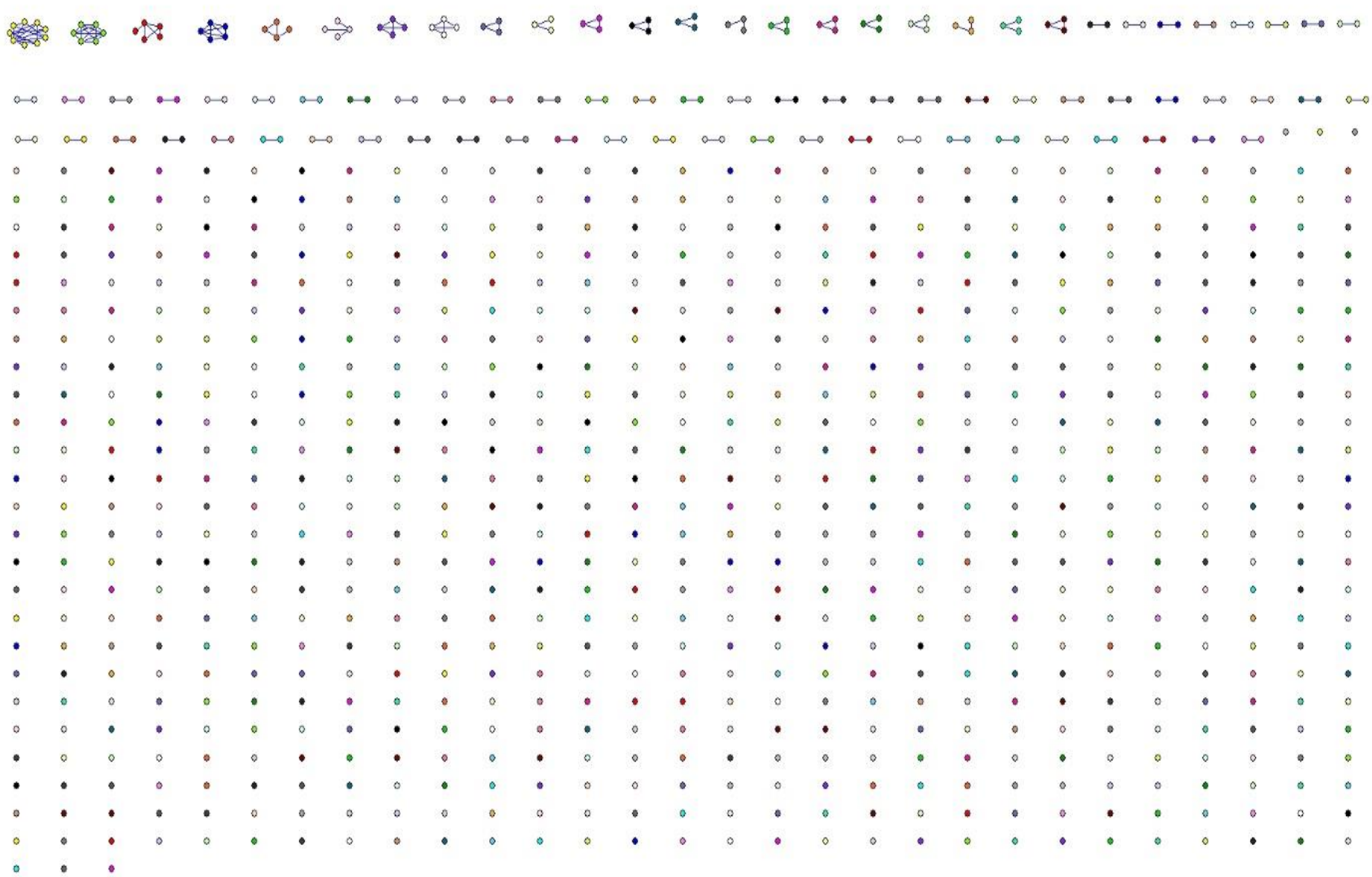

Supplement: Supplementary file 6 — Additional file 6: Figure S3.: Structures of the 815 CRMCs. Each node in the graphs is a CPC, and each connected graph represents a CRMC. (PDF 87 KB) [file 12864_2014_6723_MOESM6_ESM.pdf]

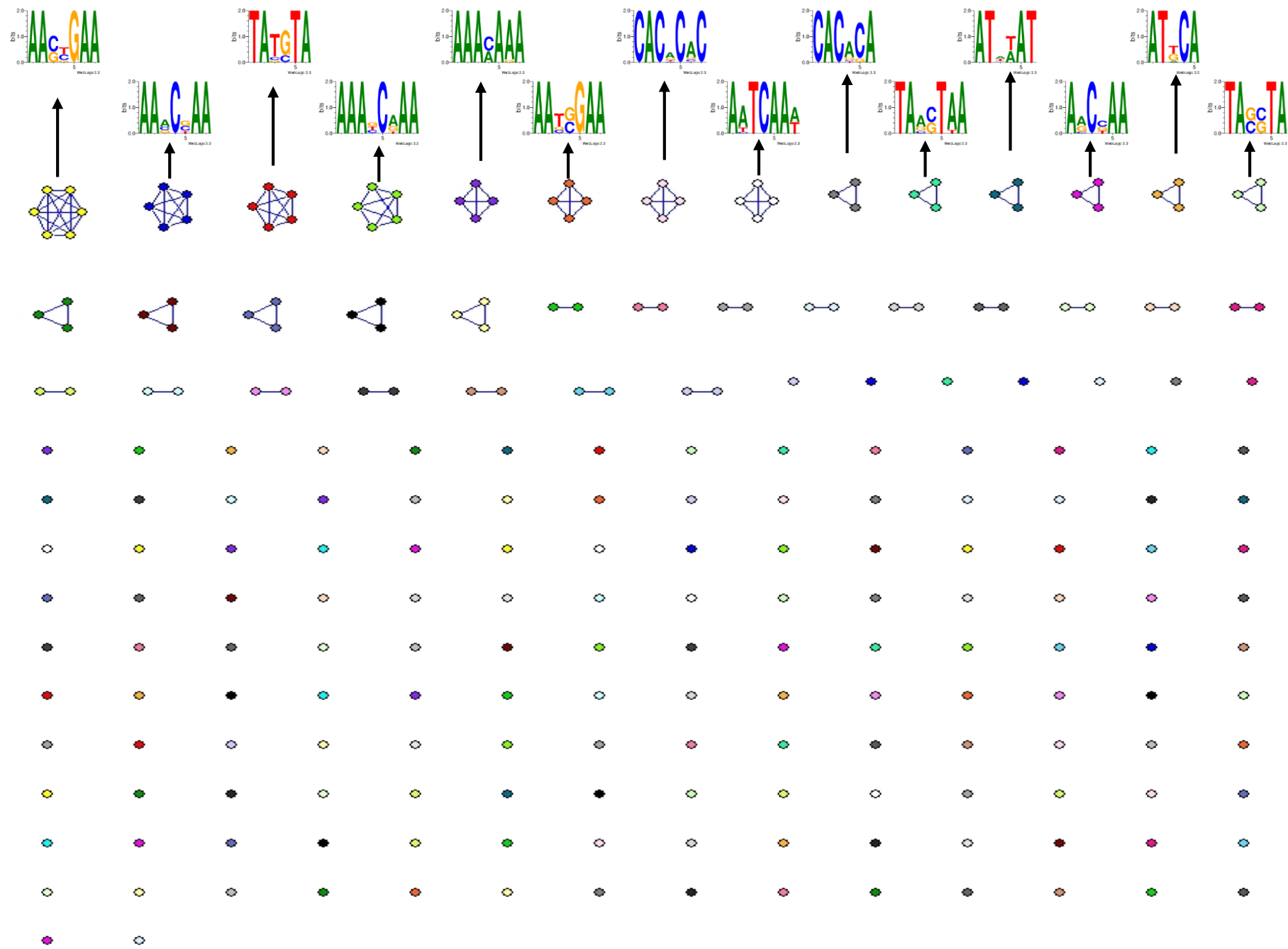

Supplement: Supplementary file 7 — Additional file 7: Figure S4.: Structures of the 184 Umotifs containing more than two motifs. Each node in the graphs is a putative motif, and each connected graph represents a Umotif. The logos are for the indicated Umotifs. (PDF 144 KB) [file 12864_2014_6723_MOESM7_ESM.pdf]

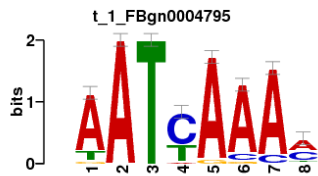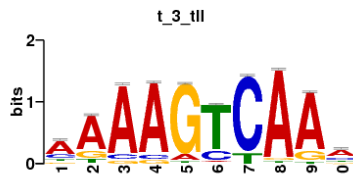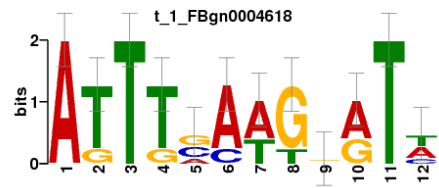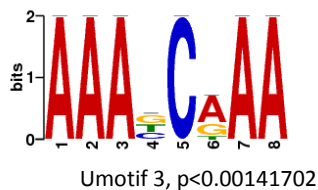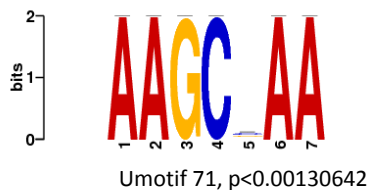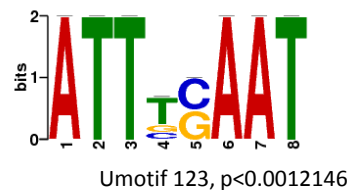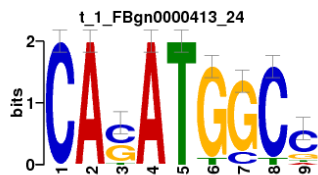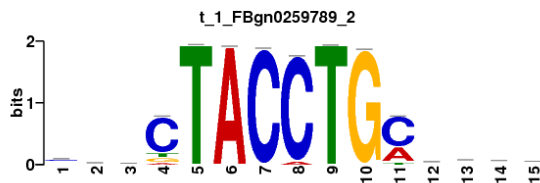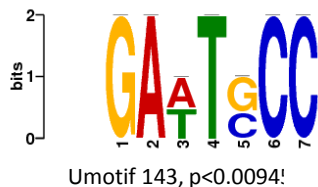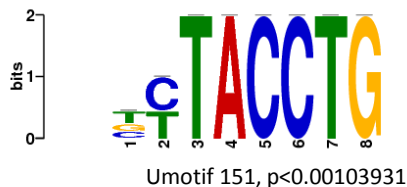

Supplement: Supplementary file 9 — Additional file 9: Figure S9.: Examples of Umotifs and their matched known motifs with a p-value around 0.001 using TOMTOM. (PDF 166 KB) [file 12864_2014_6723_MOESM9_ESM.pdf]

**A**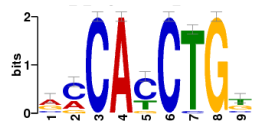

DA

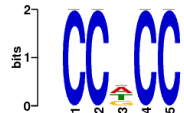

Umotif34

3014 17.55

**B**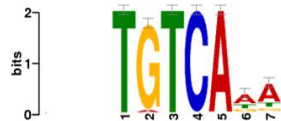

HTH

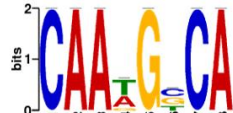

Umotif106

**C**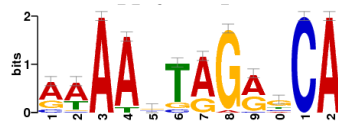

KNI

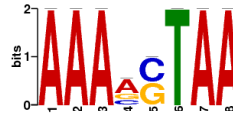

Umotif114

**D**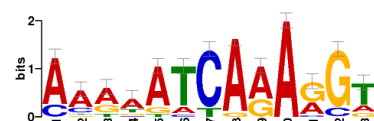

HR51

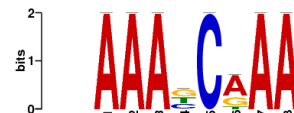

Umotif3

Torikim 14.4.2014 12:42

**E**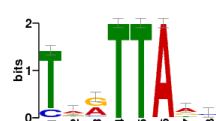

LBE

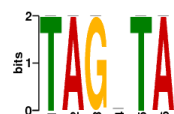

Umotif14

1502

**F**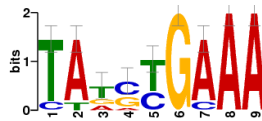

CF2

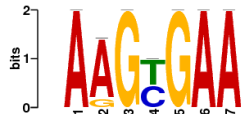

Umotif27

**G**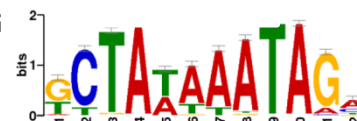

MEF2

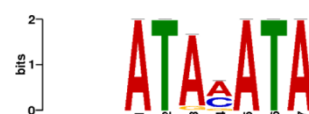

Umotif23

Supplement: Supplementary file 11 — Additional file 11: Figure S6.: Examples of known CREs in the recovered known CRMs that overlap with our predicted CREs, their corresponding Umotifs are similar the known motifs. See main text for the details. (PDF 302 KB) [file 12864_2014_6723_MOESM11_ESM.pdf]
