# Supplementary material for: De novo prediction of cis-regulatory elements and modules through integrative analysis of a large number of ChIP datasets
Source: BMC Genomics. 2014 Dec 2;15:1047. doi: 10.1186/1471-2164-15-1047 (PMC4265420; doi:10.1186/1471-2164-15-1047)
Supplement: Supplementary file 4 — Additional file 4: Figure S1.: An example of CRMs bound by TFs BCD, HB and KR from the REDfly database. The graph was shown using Gbrowser. (PDF 15 KB) [file 12864_2014_6723_MOESM4_ESM.pdf]

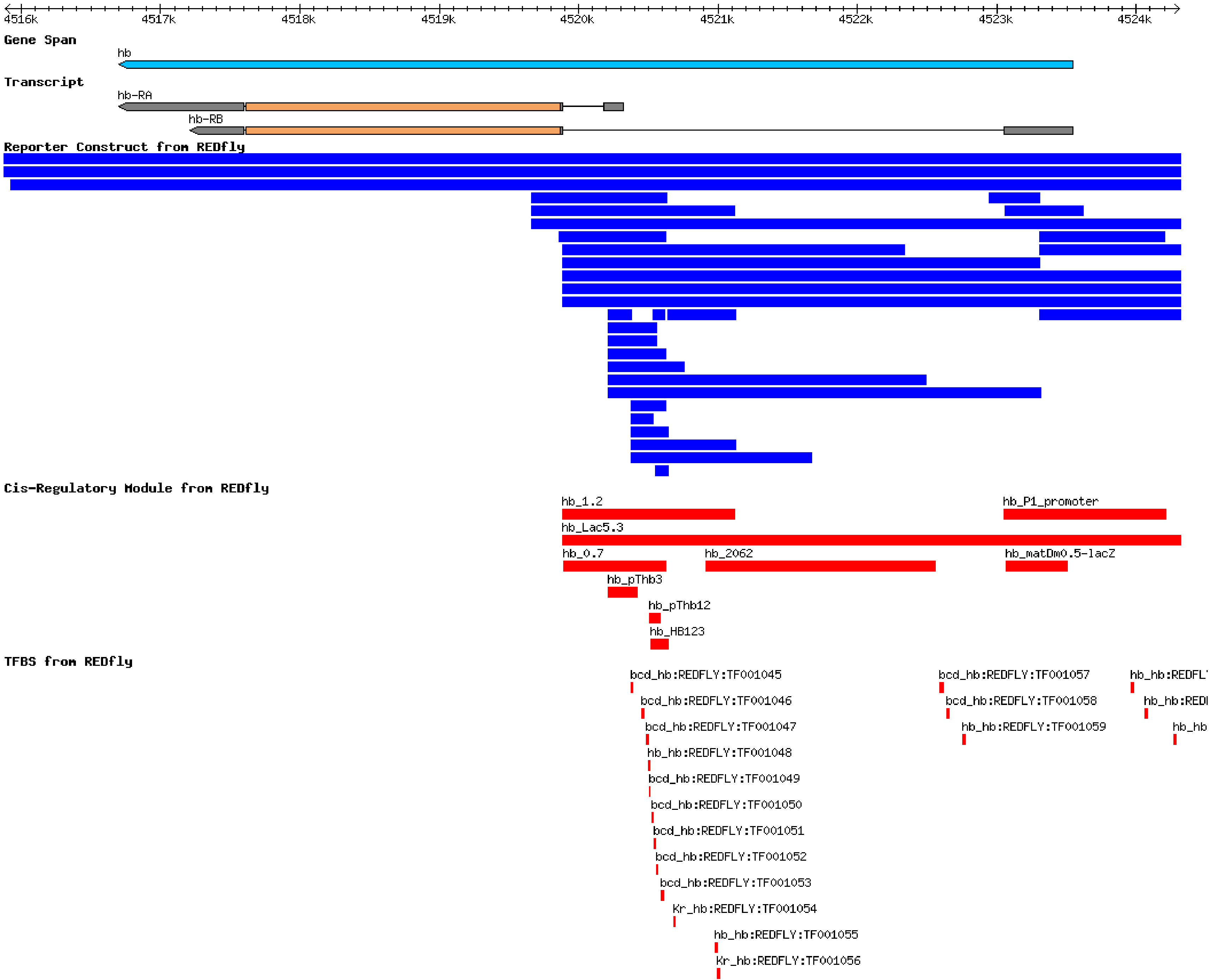

4516k
4517k
4518k
4519k
4520k
4521k
4522k
4523k
4524k
Gene Span
hb
Transcript
hb-RA
hb-RB
Reporter Construct from REDfly
Cis-Regulatory Module from REDfly
hb_1.2
hb_P1_promoter
hb_Lac5.3
hb_0.7
hb_2062
hb_matDm0.5-lacZ
hb_pThb3
hb_pThb12
hb_HB123
TFBS from REDfly
bcd_hb:REDFLY:TF001045
bcd_hb:REDFLY:TF001057
bcd_hb:REDFLY:TF001046
bcd_hb:REDFLY:TF001058
bcd_hb:REDFLY:TF001047
hb_hb:REDFLY:TF001059
hb_hb:REDFLY:TF001048
bcd_hb:REDFLY:TF001049
bcd_hb:REDFLY:TF001050
bcd_hb:REDFLY:TF001051
bcd_hb:REDFLY:TF001052
bcd_hb:REDFLY:TF001053
Kr_hb:REDFLY:TF001054
hb_hb:REDFLY:TF001055
Kr_hb:REDFLY:TF001056
