# Supplementary material for: De novo prediction of cis-regulatory elements and modules through integrative analysis of a large number of ChIP datasets
Source: BMC Genomics. 2014 Dec 2;15:1047. doi: 10.1186/1471-2164-15-1047 (PMC4265420; doi:10.1186/1471-2164-15-1047)
Supplement: Supplementary file 10 — Additional file 10: Figure S5.: A. Umotif 72 and its four individual constituent motifs found in different datasets. Umotif 72 is similar to known motifs CG12287 and CG34395. B. Umotif 27 and its five individual constituent motifs. Umotif 27 is similar to known motif CG5249. C. Umotif 70 and its four individual constituent motifs found in different datasets. D. Umotif 93 and its two individual constituent motifs found in different datasets. (PDF 277 KB) [file 12864_2014_6723_MOESM10_ESM.pdf]

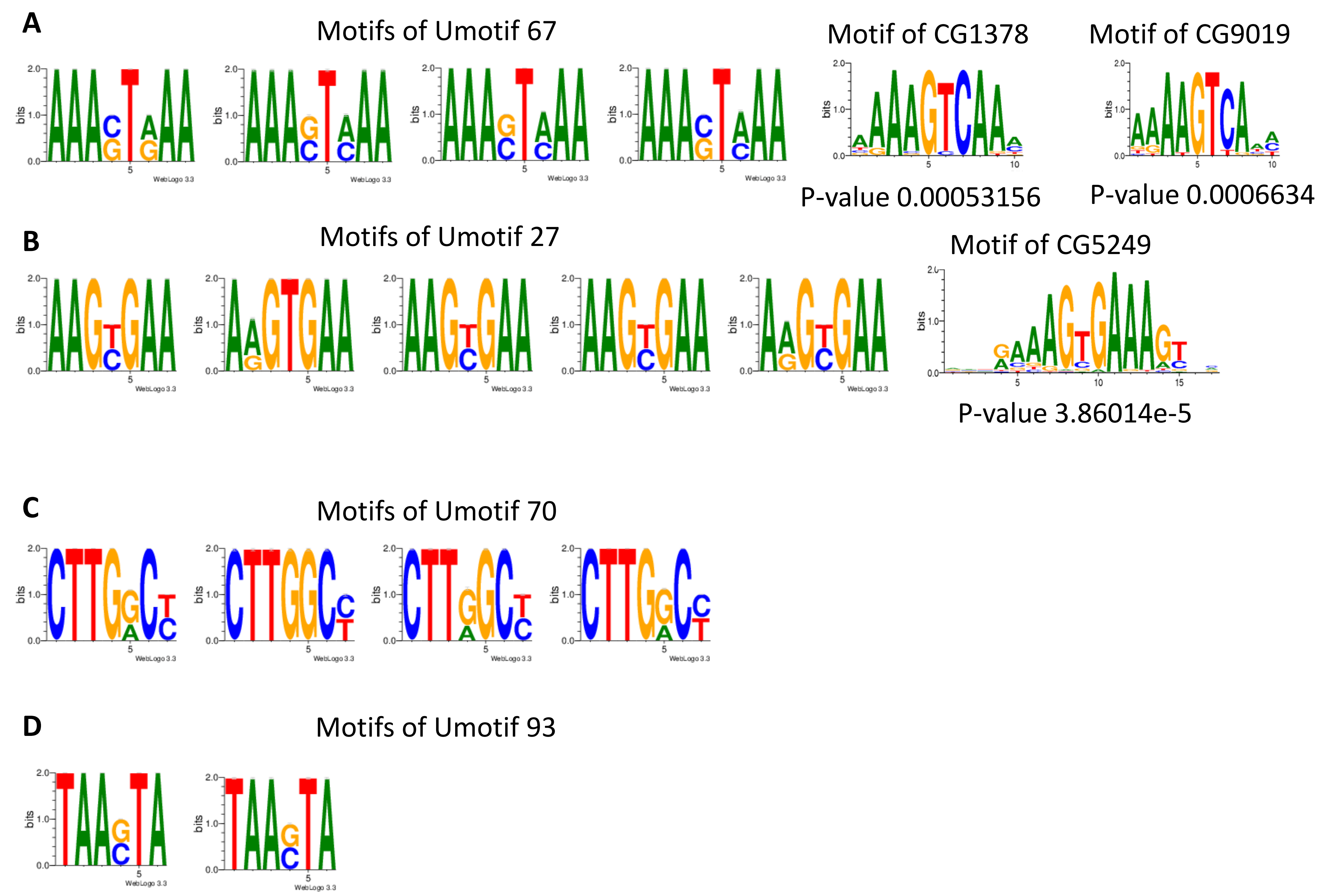
A
Motifs of Umotif 67
Motif of CG1378
Motif of CG9019
P-value 0.00053156
P-value 0.0006634
B
Motifs of Umotif 27
Motif of CG5249
P-value 3.86014e-5
C
Motifs of Umotif 70
D
Motifs of Umotif 93
