# Supplementary material for: De novo prediction of cis-regulatory elements and modules through integrative analysis of a large number of ChIP datasets
Source: BMC Genomics. 2014 Dec 2;15:1047. doi: 10.1186/1471-2164-15-1047 (PMC4265420; doi:10.1186/1471-2164-15-1047)
Supplement: Supplementary file 12 — Additional file 12: Figure S7.: A putative CRM (shown in gray shadow) is located in the first intron of gene act57B. (PDF 180 KB) [file 12864_2014_6723_MOESM12_ESM.pdf]

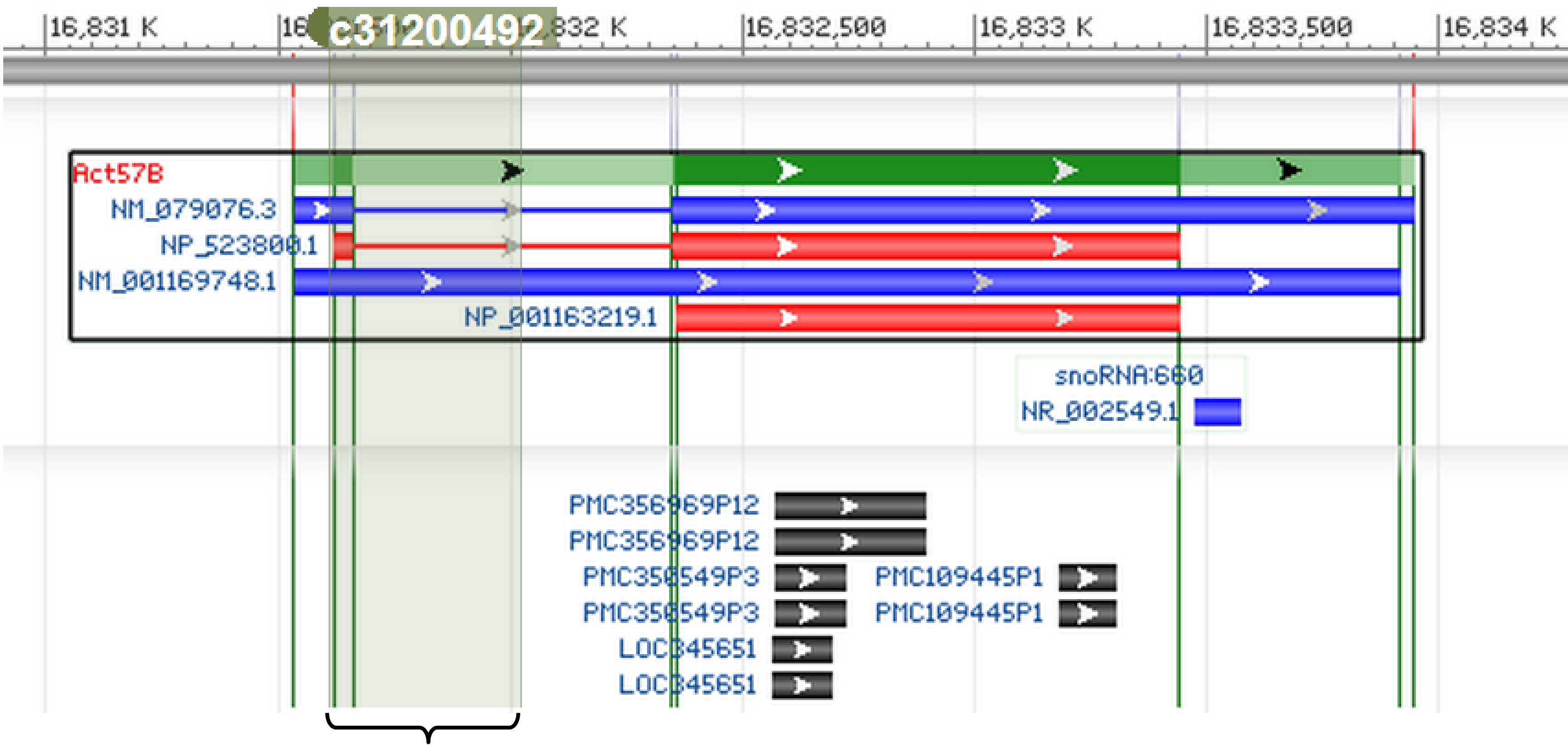

Predicted CRM
2R:16831599..16832019
